# Ganglioside Profiling of the Human Retina: Comparison with Other Ocular Structures, Brain and Plasma Reveals Tissue Specificities

**DOI:** 10.1371/journal.pone.0168794

**Published:** 2016-12-20

**Authors:** Estelle Sibille, Olivier Berdeaux, Lucy Martine, Alain M. Bron, Catherine P. Creuzot-Garcher, Zhiguo He, Gilles Thuret, Lionel Bretillon, Elodie A. Y. Masson

**Affiliations:** 1 Centre des Sciences du Goût et de l'Alimentation, CNRS, INRA, University Bourgogne Franche-Comté, Dijon, France; 2 Department of Ophthalmology, University Hospital, Dijon, France; 3 Laboratory for Biology, Imaging, and Engineering of Corneal Grafts, EA2521, Faculty of Medicine, University Jean Monnet, Saint-Etienne, France; 4 Institut Universitaire de France, Paris, France; Bascom Palmer Eye Institute, UNITED STATES

## Abstract

Gangliosides make a wide family of glycosphingolipids, highly heterogeneous in both the ceramide moiety and the oligosaccharide chain. While ubiquitously expressed in mammalian tissues, they are particularly abundant in the brain and the peripheral nervous system. Gangliosides are known to play a crucial role in the development, maintenance and functional integrity of the nervous system. However, the expression and roles of gangliosides in the retina, although often considered as a window on the brain, has been far less studied. We performed an in-depth analysis of gangliosides of the human retina, especially using powerful LC/MS methods. We compared the pattern of ganglioside classes and ceramide molecular species of this tissue with other ocular structures and with brain and plasma in elderly human individuals. About a hundred of ganglioside molecular species among 15 distinct classes were detected illustrating the huge structural diversity of these compounds. The retina exhibited a very diverse ganglioside profile and shared several common features with the brain (prominence of tetraosylgangliosides, abundance of d20:1 long chain base and 18:0 fatty acid…). However, the retina stood out with the specific expression of GD3, GT3 and AcGT3, which further presented a peculiar molecular species distribution. The unique ganglioside pattern we observed in the human retina suggests that these ganglioside species play a specific role in the structure and function of this tissue. This lipidomic study, by highlighting retina specific ganglioside species, opens up novel research directions for a better understanding of the biological role of gangliosides in the retina.

## Introduction

**Fig 1:**
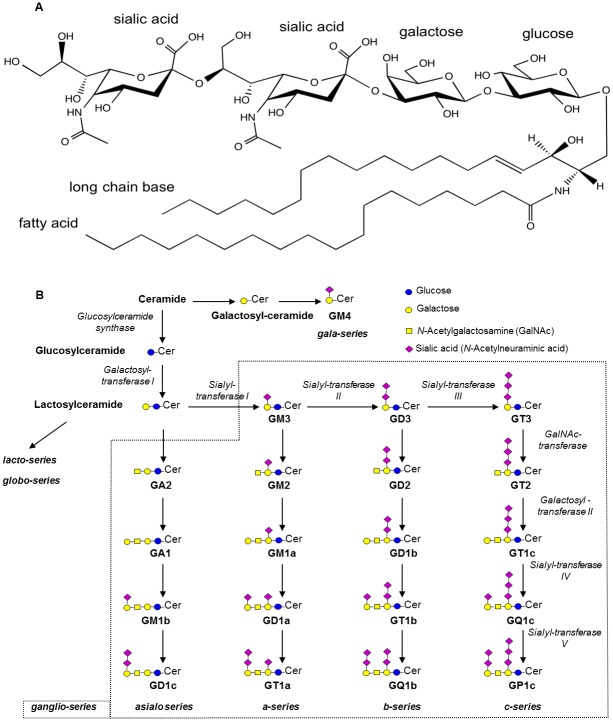
Structure of gangliosides and general scheme of biosynthesis (after Masson *et al*. [1]). A. GGs are made of a ceramide moiety composed of a long chain base (LCB) and a fatty acid (FA) whose alkyl chains may vary, and on which an oligosaccharide chain is branched. GD3 34:1 (based on LIPID MAPS, systematic name: NeuAcα2-8NeuAcα2-3Galβ1-4Glcβ-Cer) is given as an example. The ceramide is composed of sphingosine (d18:1) as LCB and stearic acid (18:0) as FA. The oligosaccharide chain is made of one glucose, one galactose and two sialic acid residues. B. The formation of GGs is catalyzed by the sequential action of glycosyltransferases (*italic*). The main GG classes of the ganglio-series are represented with their common names, according to Svennerholm [2].

Gangliosides (GGs) are glycosphingolipids making a wide family of sialic acid-containing compounds. They are composed of a ceramide moiety on which an oligosaccharide chain is branched (Fig 1A).

The ceramide is itself composed of a fatty acid (FA) linked to a sphingoid base also called long chain base (LCB). Glycosphingolipids are classified into a number of series based on their core carbohydrate structure. Most of the GGs belong to the ganglio-series characterized by the following sequence: Galβ1-3GalNAcβ1-4Galβ1-4Glcβ1-Cer [3]. Ganglio-series GGs having 0, 1, 2 and 3 sialic acid residue(s) linked to the inner galactose residue respectively make the 0-, a-, b- and c-series. As illustrated in Fig 1B, GGs are synthesized by sequential glycosylations and sialylations from the common lactosylceramide precursor. The huge diversity of GGs results from both the structural diversity of the oligosaccharide chain and the ceramide moiety. So far, more than 180 GGs have been described presenting with differences in the nature, order and linkage of the carbohydrate residues [4]. The FA and LCB also largely vary in their length, saturation and hydroxylation. Interestingly, the GG pattern differs among the tissues and cell types. It also changes during differentiation or development, as well as in some diseases.

GGs are amphiphatic molecules of the plasma membrane. They are inserted into the outer leaflet through their hydrophobic ceramide moiety and expose their hydrophilic oligosaccharide chain towards the extracellular environment. They are thought to be enriched within ordered membrane microdomains referred to as lipid rafts [5, 6]. This special localization governs their biological roles. Indeed, GGs are known to be involved in glycosylation-dependent adhesion, recognition and signaling by regulating cell-cell and cell-matrix interactions. They are receptors for microorganisms and toxins and also interact with other glycosphingolipids, counterpart lectins and adhesion receptors such as integrins. Moreover, GGs have been shown to modulate transmembrane signaling via two major systems: growth factor receptor-associated tyrosine kinase and PKC. Through these functions, GGs can regulate cell proliferation and death, adhesion, migration and differentiation [7].

While ubiquitously expressed in mammalian tissues, GGs are particularly abundant in the brain and nervous tissues. Their importance in the nervous system is now well established. *In vitro*, GGs have been associated with neuritogenesis, survival and adhesion of neural cells [7]. *In vivo*, GG expression undergoes dramatic changes during neurodevelopment, especially during the early stages suggesting their functional roles at particular developmental milestones such as neurogenesis and synaptogenesis [8]. In the last 20 years, several strain lines of knock-out mice for GG biosynthesis enzymes (GM3 synthase, GD3 synthase, or GM2/GD2 synthase) have been established and their phenotype characterized. These mice show varying degrees of neurological disorders aggravating with age such as motor and sensory dysfunctions, impaired nerve regeneration, demyelination and inflammation of neurons and behavioural deficits… [8–10]. In humans, a group of diseases caused by alterations of the degradation of GGs, which consecutively accumulate into lysosomes, have been described. They are referred to as gangliosidosis (GM2-gangliosidoses: Tay-Sachs and Sandhoff diseases or GM1-gangliosidosis). Although the onset, course and clinical manifestations may vary, they are marked by severe neurodegenerative disorders (developmental arrest, seizures, dystonia, hypotonia and dementia) [11]. It is noticeable that most of them also exhibit visual symptoms ranging from a cherry red spot in the central retina to loss of vision. Moreover, Simspon *et al*. reported the association of a loss-of-function mutation of GM3 synthase leading to a lack of ganglio-series GG expression with an epilepsy syndrome, developmental stagnation and blindness among the Amish people [12].

Several studies reported important developmental changes in GG composition of the retina, concomitantly to morphological differentiation, especially in chick embryos [13–15]. GD3 undergoes a drastic decrease while the more complex GGs, especially GM1 and GD1a, increase to become the major classes during the first 15 embryonic days when synaptogenesis, growth of the outer segment of the photoreceptors and emergence of a bioelectric response to light stimulation happen. While the decrease in GD3 levels also occurs in mammalian retina, this GG class remains prevalent at all developmental stages, as opposed to the brain in which tetraosylGGs become the major classes [16–19]. Characterization of mouse models of Sandhoff disease revealed that they exhibit an impaired capability of neurite outgrowth in retinal ganglion cells [20] and visual impairment [21]. Besides, a few studies suggested that GGs exert a neuroprotective action within the retina. It was shown that GM1 injection into the vitreous inhibited the degeneration of retinal ganglion cells after optic nerve axotomy [22] or protected against retinal damage (retinal thickness atrophy, decrease in retinal ganglion cell density) induced by retinal ischemia [23]. While those studies strongly suggest a major role of GGs in the retina, this research field remains poorly investigated and requires further work to improve the comprehension of the precise roles of GGs in retina’s structure and function.

In the present work, we analyzed GGs in the human retina and other nervous and non-nervous ocular structures (optic nerve, retinal pigment epithelium (RPE)/choroid, ciliary body) of aged individuals. We also analyzed GGs in the brain, whose retina is a direct extension, and in plasma where GGs are circulating associated with lipoproteins. By comparing the GG profiles of these different tissues of interest, we aimed to highlight specificities and/or common features in order to give insight regarding the specific roles of GGs in the structure and function of the retina. This work especially involved LC/MS techniques that offer a sensitive, accurate and detailed picture of GGs and their ceramide profile.

## Materials and Methods

### Ethics statement

All fresh tissues were procured from bodies donated to science (Laboratory of Anatomy, Faculty of Medicine of St-Etienne, France) as permitted by the French bioethics law. Donors volunteer their body and give written consent to the Laboratory of Anatomy; no further specific approval by the ethics committee is required. Handling of donor tissues adhered to the tenets of the Declaration of Helsinki of 1975 and its 1983 revision in protecting donor confidentiality.

### Materials

Chloroform (CHCl_3_) and methanol (CH_3_OH) were obtained from SDS, France. Ammonium acetate, acetonitrile (CH_3_CN), methanol and water (H_2_O) of Optima LC/MS grade were all from Fisher Scientific, France. Commercially available GG standards (GM3, GM2, GM1, GD3, GD1a, GD1b, GT1b and GQ1b) from natural sources (bovine or human) were obtained from Matreya LLC, USA.

### Human tissues

Human tissues were obtained from 7 donors (5 women and 2 men) ranging from 81 to 94 years old (mean age 87.3 ± 5.9 years). Bodies were refrigerated at 4°C and tissues collected within 24 h after death (mean time 14.0 ± 5.7 hours). A blood sample was collected in heparinized tubes by venipuncture. Plasma was separated from red blood cells by centrifugation at 250 *g* for 10 min at 4°C. After removing the eyeballs, a brain biopsy was performed through the orbit roof (in the area of the frontal lobe). After removing the anterior part of the eye, the ciliary body was identified anterior to the pars plana and carefully dissected. The posterior pole of the eyeball was placed on a back-lit table and the retina was observed under an operating microscope. No eye having large drusen, severe pigment epithelial alterations, severe macular atrophy, macular hemorrhage, or any grossly visible chorioretinal pathologic abnormality was included in the study. Moreover, a gross examination of the optic nerve head did not reveal any case of glaucoma within the donors. After removing the vitreous body, the entire neural retina was carefully separated from the RPE/choroid with forceps. The optic nerve was then excised by cutting tangential to the sclera after having removed the extraocular tissues. All samples were immediately stored at -80°C until further analyses.

### Tissue sample preparation

50–300 mg of tissue (retina, optic nerve, RPE/choroid, ciliary body, brain) was thoroughly homogenized in 0.5–1 mL distilled H_2_O with tungsten microbeads using a tissue lyser (Qiagen, The Netherlands, 1 min 30 s at 30 Hz speed). A 40 μL aliquot of each sample was used to measure protein content. The rest of the sample was used to extract gangliosides. Alternatively, 1–5 mL of plasma were used. A 40 μL aliquot of each sample was also used to measure protein content.

### Quantitative determination of protein content

The 40 μL aliquots of tissue homogenates and plasma were lysed by addition of 10 μL of 5X Ripa buffer and incubation on ice for 30 min. After clearing by centrifugation (10 000 *g*, 10 min, 4°C), total protein content was measured on the supernatants using the BCA method (BCA protein assay kit according to manufacturer’s instructions, Pierce Biotechnology, Thermo Scientific, USA) with bovine serum albumin as a standard (Sigma-Aldrich, USA).

### Extraction, separation and purification of gangliosides

First, total lipids were extracted from the tissue samples homogenized in H_2_O and from plasma, overnight at 4°C, with 10 volumes of CHCl_3_/CH_3_OH (1:1, v/v). The residual pellets obtained after centrifugation (1 500 *g*, 5 min) were then re-extracted with 3 mL CHCl_3_/CH_3_OH (1:1, v/v), 3 mL CHCl_3_/CH_3_OH (2:1, v/v) and 3 mL CHCl_3_/CH_3_OH/H_2_O (48:35:10, v/v/v). The four lipid extracts were combined, dried under a stream of nitrogen and re-dissolved in 3 mL CHCl_3_/CH_3_OH (1:1, v/v). Second, GG were separated from other lipids by phase partition by adding 1 mL H_2_O. After centrifugation (1 000 *g*, 3 min), the upper aqueous phases were collected while lower organic phases were re-extracted twice with 2 mL CH_3_OH/H_2_O (1:1, v/v). The three upper phases containing GGs were combined, dried under a stream of nitrogen and re-dissolved in 2 mL CH_3_OH/PBS 10mM (1:1, v/v). Third, the GG extracts were desalted on a C18 silica gel column (Sep-Pak Vac 6cc, 500 mg, Waters, USA) washed with 7 mL CH_3_OH and pre-equilibrated with 7 mL CH_3_OH/PBS 10mM (1:1, v/v). After washing with 10 mL H_2_O, purified GGs were eluted with 6 mL CH_3_OH and 4 mL CHCl_3_/CH_3_OH (2:1, v/v). GG extracts were evaporated to dryness under a stream of nitrogen, re-dissolved in 1 mL CHCl_3_/CH_3_OH (2:1, v/v) and stored, under nitrogen, at -20°C until further analyses.

### Ganglioside analysis by High Performance Thin Layer Chromatography (HPTLC)

Aliquots of the GG extracts (equivalent to 10 nmol GG-bound sialic acid as a pool of the individual samples for each tissue type, n = 4–7) were applied to a HPTLC plate (Merck, Germany). The plate was developed in CHCl_3_/CH_3_OH/0.2% CaCl_2_ (55:45:10, v/v/v) for 45 min, sprayed with resorcinol reagent (10 mL resorcinol 2% (Sigma-Aldrich, USA), 40 mL hydrochloric acid, and 0.125 mL 0.1M copper sulfate) and covered with a clean glass plate to be heated at 120°C for 20 min. GGs, appearing as purple-blue bands, were identified by co-migration with standards. A standard curve was also performed for each GG class by spotting increasing known amounts of GG standards on a HPTLC plate. A densitometric analysis of the GG-bound sialic acid detected on the plates was performed, using a CCD camera (Chemidoc^™^ MP System from Biorad, USA). Tissue GGs were then quantified using the standard curves established for each GG class.

### Ganglioside analysis by liquid chromatography coupled to mass spectrometry

GGs were first separated by liquid chromatography under Hydrophilic Interaction Liquid Chromatography (HILIC) conditions and then analyzed by both a Triple Quadrupole mass spectrometer and a LTQ Orbitrap mass spectrometer thanks to analytical methods previously developed. For a precise description of the methods, see [1].

### High performance liquid chromatography

GG separation was achieved using a silica Kinetex column (150 mm x 2.1 mm i.d., 2.6 μm, HILIC, Phenomenex, USA) maintained at 30°C. The mobile phase consisted of (A) CH_3_CN/H_2_O (90:10, v/v) containing 10 mM ammonium acetate and (B) CH_3_CN/H_2_O (50:50, v/v) containing 10 mM ammonium acetate. The solvent-gradient system was as follows: 0–1 min 100% A, 4 min 79% A, 9 min 78% A, 14–18 min 50% A and 19–48 min 100% A.

### TSQ quantum ultra

The triple quadrupole (QqQ) mass spectrometer (Thermo Scientific, USA) was operated in negative ion mode. For characterization, Collision-Induced Dissociation (CID) of each deprotonated molecule was performed in the negative mode. An abundant product ion at *m/z* 290 corresponding to a characteristic *N*-Acetylneuraminic acid (sialic acid) fragment was obtained from [M-xH]^x-^ ions of the different GG molecular species. This fragment was used for precursor ion scanning, whereby the [M-xH]^x-^ ions of GG were specifically detected (Signal/Noise > 3). For quantification, data were acquired in Selected Reaction Monitoring (SRM). The precursor and product ion pairs for the SRM analysis were selected based on the precursor ion scanning but some species were not considered in SRM because they stood below the quantification limit (Signal/Noise < 10). Each sample was injected in triplicate. The proportion of each molecular species of a specific GG class was calculated as the ratio of its peak area to the sum of all detected peak areas in this class, every GG class being considered separately.

### Thermo LTQ-Orbitrap XL^™^ (Thermo Scientific, USA)

This hybrid mass spectrometer was used for high-resolution analyses. All spectra were acquired in the mass range *m/z* 200–2600 and with the resolution set value 60 000 at *m/z* 400. For tandem mass spectrometry (MS/MS) analyses, Higher energy Collisional Dissociation (HCD) and CID were both employed. The precursor ions were selected within an isolation width of 10.

### Statistical analyses

For each tissue and each GG class, intensity measurements of molecular species obtained with the QqQ mass spectrometer were expressed as proportions. First, averages over the three replicated injections were calculated. Subsequently, averages over subjects (n = 7 for retina, brain and RPE/choroid, n = 6 for optic nerve, n = 4 for ciliary body and plasma) were represented in colorscale. Those species proportions represent compositional data which were further analyzed using the R package ‘compositions’ (K. Gerald van den Boogaart, Raimon Tolosana and Matevz Bren (2014).compositions: Compositional Data Analysis. R package version 1.40–1. https://CRAN.R-project.org/package=compositions). The analyses focused on several variables selected for their biological relevance: the proportion of (36:1 + 38:1) ceramides, of 34:1 ceramide, of (40:1 + 42:2 + 42:1) ceramides, of ceramides with 1 or 2 unsaturation(s), of ceramides with 34 carbons or less and of ceramides with 42 carbons or more. Those proportions were obtained using the real compositional scale (rcomp) since the high number of “below detection limit” values made impossible the utilization of Aitchison compositional scale. Each variable was transformed using a centered log ratio transformation. For each variable, analyses of variance were performed by GG class, with an additive model with tissue and subject factors. Then, tissues were compared through Newman-Keuls multiple comparisons (Table 1). Furthermore, ternary diagrams, suited for the representation of composition and relative data, were plotted for the retina and for retinal and brain GT3 and AcGT3. They represent the proportions of several variables: (36:1 + 38:1), (40:1 + 42:2 + 42:1) ceramides and other molecular species; ceramides with 1, 2 and 3 unsaturations; ceramides with 34 carbons or less, 42 carbons or more and other molecular species, as well as confidence ellipses. For all statistical analyses, significance was set at p<0.05.

**Table 1:** Ceramide proportions in the GG classes of the retina and other ocular tissues, brain and plasma.

	**A. (36:1+38:1) ceramides**						**B. 34:1 ceramides**					
**GG classes**	*Retina*	*Brain*	*RPE / Choroid*	*Ciliary Body*	*Optic nerve*	*Plasma*	*Retina*	*Brain*	*RPE / Choroid*	*Ciliary Body*	*Optic nerve*	*Plasma*
**GM3**	0.05^d^	0.04^d^	0.19^b^	0.17^b^	0.08^c^	0.37^a^	0.61^b^	0.71^a^	0.26^c^	0.17^d^	0.17^d^	0.14^e^
**GM2**	0.03^b^	0.03^b^	N.D.	0.26^a^	0.01^c^	0.25^a^	0.95^a^	0.91^a^	N.D.	0.41^b^	0.81^a^	0.32^b^
**GD3**	0.06^c^	0.02^e^	0.20^b^	0.23^b^	0.05^d^	0.39^a^	0.54^b^	0.84^a^	0.24^cd^	0.18^d^	0.31^c^	0.10^e^
**AcGD3**	0.06^b^	0^d^	N.D.	0.32^a^	0.03^c^	N.D.	0.66^b^	0.65^b^	N.D.	0.12^c^	0.85^a^	N.D.
**GD2**	0.01^b^	0.01^bc^	N.D.	0.28^a^	0^d^	N.D.	0.96^a^	0.95^a^	N.D.	0.66^a^	1.00^a^	N.D.
**GD1a**	0.03^d^	0.01^e^	0.04^c^	0.17^b^	0^e^	0.39^a^	0.87^a^	0.89^a^	0.16^d^	0.28^c^	0.38^b^	0.38^b^
**GD1b**	0.01^bc^	0.01^c^	0.01^bc^	0.20^a^	0^d^	0.04^b^	0.90^a^	0.92^a^	0.86^a^	0.70^b^	0.69^b^	0.84^a^
**AcGD1b**	0^a^	0^a^	N.D.	N.D.	0^a^	N.D.	0.90^a^	0.99^a^	N.D.	N.D.	1.00^a^	N.D.
**GT3**	0.04^a^	0^b^	N.D.	N.D.	N.D.	N.D.	0.34^b^	0.98^a^	N.D.	N.D.	N.D.	N.D.
**AcGT3**	0.02^a^	0^b^	N.D.	N.D.	N.D.	N.D.	0.38^b^	1.00^a^	N.D.	N.D.	N.D.	N.D.
**GT1b**	0.02^b^	0.01^bc^	0.01^b^	0.16^a^	0^c^	0.11^a^	0.85^a^	0.96^a^	0.76^a^	0.54^b^	0.57^b^	0.60^b^
**AcGT1b**	0^b^	0^b^	0^b^	0.26^a^	0^b^	N.D.	0.94^a^	0.96^a^	N.D.	0.32^b^	0.86^a^	N.D.
**GQ1b**	0^b^	0^b^	0^b^	0.20^a^	0^b^	N.D.	0.85^a^	0.93^a^	1.00^a^	0.69^a^	0.74^a^	N.D.
**AcGQ1b**	0^a^	0^a^	N.D.	N.D.	0^a^	N.D.	1.00^a^	1.00^a^	N.D.	N.D.	0.93^b^	N.D.
	**C. (40:1+42:2+42:1) ceramides**						**D. Ceramides with 1 unsaturation**					
**GG classes**	*Retina*	*Brain*	*RPE / Choroid*	*Ciliary Body*	*Optic nerve*	*Plasma*	*Retina*	*Brain*	*RPE / Choroid*	*Ciliary Body*	*Optic nerve*	*Plasma*
**GM3**	0.85^a^	0.84^a^	0.69^c^	0.65^d^	0.61^e^	0.72^b^	0.21^d^	0.19^d^	0.43^b^	0.46^b^	0.60^a^	0.27^c^
**GM2**	0.99^a^	0.96^a^	N.D.	0.83^c^	0.89^b^	0.71^d^	0.01^d^	0.02^c^	N.D.	0.30^a^	0.15^b^	0.27^a^
**GD3**	0.87^a^	0.91^a^	0.70^b^	0.71^b^	0.70^b^	0.67^b^	0.32^c^	0.10^d^	0.46^a,b^	0.41^b^	0.53^a^	0.30^c^
**AcGD3**	0.93^b^	0.70^d^	N.D.	0.76^c^	0.99^a^	N.D.	0.22^b^	0.05^d^	N.D.	0.48^a^	0.10^c^	N.D.
**GD2**	0.99^a^	0.98^a^	N.D.	0.97^a^	1.00^a^	N.D.	0.02^c^	0.03^b^	N.D.	0.06^a^	0^d^	N.D.
**GD1a**	0.93^a^	0.93^a^	0.65^d^	0.69^c^	0.68^c^	0.84^b^	0.03^f^	0.05^e^	0.75^a^	0.42^b^	0.33^c^	0.08^d^
**GD1b**	0.98^a^	0.97^a^	0.96^a^	0.95^a^	0.94^a^	0.95^a^	0.07^b^	0.05^b^	0.12^b^	0.08^b^	0.27^a^	0.10^b^
**AcGD1b**	0.93^a^	0.99^a^	N.D.	N.D.	1.00^a^	N.D.	0.03^a^	0^b^	N.D.	N.D.	0^b^	N.D.
**GT3**	0.81^b^	0.98^a^	N.D.	N.D.	N.D.	N.D.	0.54^a^	0^b^	N.D.	N.D.	N.D.	N.D.
**AcGT3**	0.80^b^	1.00^a^	N.D.	N.D.	N.D.	N.D.	0.52^a^	0^b^	N.D.	N.D.	N.D.	N.D.
**GT1b**	0.96^ab^	0.98^a^	0.93^ab^	0.85^c^	0.90^bc^	0.85^c^	0.11^b^	0.01^c^	0.21^a^	0.25^a^	0.35^a^	0.24^a^
**AcGT1b**	1.00^a^	0.99^a^	0^b^	0.65^a^	1.00^a^	N.D.	0.06^ab^	0.04^b^	0^c^	0.17^ab^	0.14^a^	N.D.
**GQ1b**	0.97^bc^	0.97^c^	1.00^a^	0.98^b^	0.97^bc^	N.D.	0.13^b^	0.04^d^	0^e^	0.10^c^	0.26^a^	N.D.
**AcGQ1b**	1.00^a^	1.00^a^	N.D.	N.D.	1.00^a^	N.D.	0^b^	0^b^	N.D.	N.D.	0.07^a^	N.D.
	**E. Ceramides with 2 unsaturations**						**F. Ceramides ≤ 34 carbons**					
**GG classes**	*Retina*	*Brain*	*RPE/ Choroid*	*Ciliary Body*	*Optic nerve*	*Plasma*	*Retina*	*Brain*	*RPE/ Choroid*	*Ciliary Body*	*Optic nerve*	*Plasma*
**GM3**	0.15^d^	0.16^d^	0.31^bc^	0.35^ab^	0.39^a^	0.28^c^	0.05^d^	0.04^d^	0.20^b^	0.19^b^	0.08^c^	0.44^a^
**GM2**	0.01^e^	0.04^d^	N.D.	0.17^b^	0.11^c^	0.29^a^	0.03^b^	0.03^b^	N.D.	0.26^a^	0.01^c^	0.27^a^
**GD3**	0.13^b^	0.09^c^	0.30^a^	0.29^a^	0.30^a^	0.33^a^	0.06^c^	0.02^e^	0.21^b^	0.25^b^	0.05^d^	0.48^a^
**AcGD3**	0.07^b^	0.30^a^	N.D.	0.24^a^	0.01^c^	N.D.	0.06^b^	0^d^	N.D.	0.32^a^	0.03^c^	N.D.
**GD2**	0.01^b^	0.02^a^	N.D.	0.03^a^	0^c^	N.D.	0.01^b^	0.01^bc^	N.D.	0.28^a^	0^c^	N.D.
**GD1a**	0.07^c^	0.07^c^	0.35^a^	0.31^a^	0.32^a^	0.16^b^	0.03^d^	0.01^e^	0.04^c^	0.18^b^	0^e^	0.46^a^
**GD1b**	0.02^a^	0.03^a^	0.04^a^	0.05^a^	0.06^a^	0.05^a^	0.01^bc^	0.01^c^	0.01^bc^	0.20^a^	0^d^	0.05^b^
**AcGD1b**	0.01^a^	0.01^a^	N.D.	N.D.	0^b^	N.D.	0^a^	0^a^	N.D.	N.D.	0^a^	N.D.
**GT3**	0.19^a^	0.02^b^	N.D.	N.D.	N.D.	N.D.	0.04^a^	0^b^	N.D.	N.D.	N.D.	N.D.
**AcGT3**	0.19^a^	0^b^	N.D.	N.D.	N.D.	N.D.	0.02^a^	0^b^	N.D.	N.D.	N.D.	N.D.
**GT1b**	0.04^b^	0.02^c^	0.07^ab^	0.15^a^	0.10^a^	0.15^a^	0.02^b^	0.01^bc^	0.01^b^	0.16^a^	0^c^	0.11^a^
**AcGT1b**	0^b^	0.01^a^	0^b^	0.10^a^	0^b^	N.D.	0^b^	0^b^	N.D.	0.26^a^	0^b^	N.D.
**GQ1b**	0.03^a^	0.03^a^	0^c^	0.01^b^	0.03^a^	N.D.	0^b^	0^b^	0^b^	0.20^a^	0^b^	N.D.
**AcGQ1b**	0^a^	0^a^	N.D.	N.D.	0^a^	N.D.	0^a^	0^a^	N.D.	N.D.	0^a^	N.D.
	**G. Ceramides ≥ 42 carbons**											
**GG classes**	*Retina*	*Brain*	*RPE/ Choroid*	*Ciliary Body*	*Optic nerve*	*Plasma*						
**GM3**	0.10^e^	0.17^d^	0.37^b^	0.37^b^	0.56^a^	0.21^c^						
**GM2**	0^c^	0.01^c^	N.D.	0.23^a^	0.15^b^	0.22^a^						
**GD3**	0.18^c^	0.08^d^	0.39^a^	0.29^b^	0.43^a^	0.30^b^						
**AcGD3**	0^b^	0^b^	N.D.	0.32^a^	0^b^	N.D.						
**GD2**	0^b^	0.01^b^	N.D.	0.04^a^	0^b^	N.D.						
**GD1a**	0.01^e^	0.01^e^	0.65^a^	0.37^b^	0.23^c^	0.07^d^						
**GD1b**	0.01^b^	0.01^b^	0.06^a^	0.06^a^	0.12^a^	0.05^a^						
**AcGD1b**	0^a^	0^a^	N.D.	N.D.	0^a^	N.D.						
**GT3**	0.31^a^	0^b^	N.D.	N.D.	N.D.	N.D.						
**AcGT3**	0.29^a^	0^b^	N.D.	N.D.	N.D.	N.D.						
**GT1b**	0.03^b^	0.01^c^	0.11^a^	0.21^a^	0.21^a^	0.20^a^						
**AcGT1b**	0^b^	0^b^	0^b^	0.14^a^	0^b^	N.D.						
**GQ1b**	0.03^b^	0^c^	0^c^	0.06^a^	0.07^a^	N.D.						
**AcGQ1b**	0^a^	0^a^	N.D.	N.D.	0^a^	N.D.						

Data are presented as proportions (mean of 4–7 independent samples). Seven variables were considered: (36:1+38:1) ceramides, 34:1 ceramides, (40:1+42:2+42:1) ceramides, ceramides with 1 unsaturation, ceramides with 2 unsaturations, ceramides with 34 carbons or less and ceramides with 42 carbons or more. Letters refer to results of the Student Newman Keuls multiple comparisons of tissues. For each variable, within a GG class, the letter "a" is attributed to the highest proportions among the 6 tissues considered. Different letters indicate significant differences in the proportions between tissues (p<0.05). N.D.: Non-detected GG class.

## Results

### Total ganglioside content and ganglioside pattern determined by HPTLC

**Fig 2:**
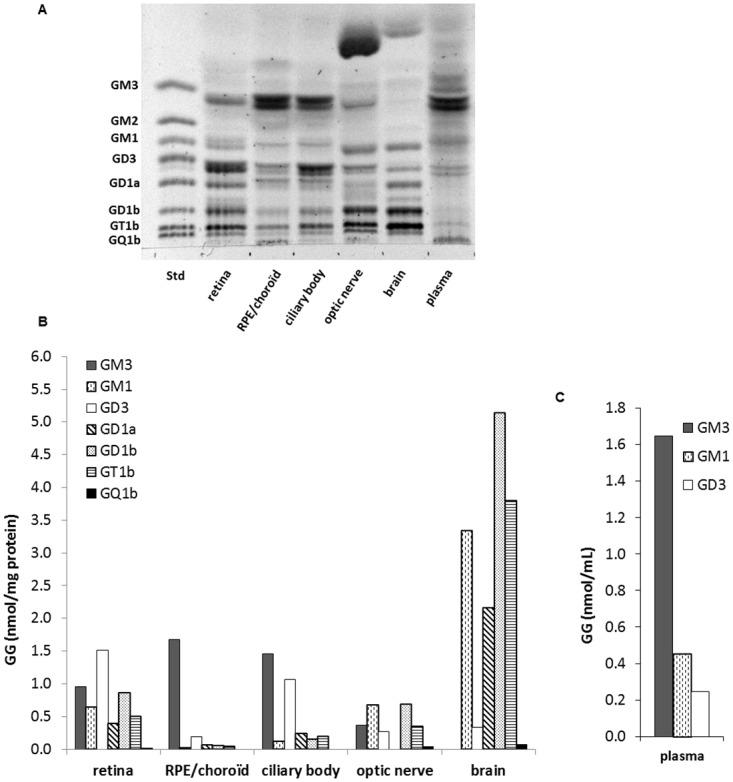
Ganglioside profile of the retina and other ocular tissues, brain and plasma. A. Resorcinol-stained HPTLC plate of GGs extracted from retina, retinal pigment epithelium (RPE)/choroid, ciliary body, optic nerve, brain and plasma. GG aliquots (4–7 independent samples) were pooled for each tissue type to represent a total of 10 nmol GG-sialic acid spotted per lane. A standard mixture of ganglio-series GGs (Std, 5 nmol sialic acid) was also spotted. The plate was developed in CHCl_3_/CH_3_OH/0.2% CaCl_2_ (55:45:10, v/v/v) and revealed with resorcinol reagent. B. Quantitative distribution of GG classes calculated from a standard curve of each GG class. Results are expressed in nmol GG/mg protein or nmol GG/mL plasma.

The GG pattern of the different tissues of interest was studied by HPTLC associated to resorcinol revelation. As shown in Fig 2A, significant discrepancies among the ocular tissues, brain and plasma clearly appeared. GGs of the ganglio-series were identified based on comigration with standards and quantitative assessment was obtained by densitometric analysis thanks to calibration curves established for each GG class. Results expressed as nmol GG/mg protein for tissues and as nmol GG/mL for plasma are given in Fig 2B and 2C, respectively.

Regarding the total GG content, the retina contained higher amounts than the other ocular structures since the total reached 4.5 nmol GG/mg protein for the retina and 2.1, 3.2 and 2.2 nmol GG/mg prot for the RPE/choroid, ciliary body and optic nerve, respectively. However, those levels were much lower than the levels measured in the brain (14.8 nmol GG/mg protein) which is known to be particularly rich in GGs. Those results are in accordance with previously published data obtained in rat retina and brain [17]. The circulating GG levels were 2.3 nmol GG/ml plasma.

Regarding the GG pattern, tissue specificities clearly appeared. The retina presented a large diversity of GGs since 7 different classes could be detected and quantified: GM3, GM1, GD3, GD1a, GD1b, GT1b and GQ1b. Retina GGs belonged to both the a- and b ganglio-series and were mono-, di-, tri- or quadrisialylated. As in other types of nervous tissues such as the brain, tetraosylGGs (GM1, GD1a, GD1b and GT1b) were present in large proportions but the prominence of GD3 was a specific feature of the retina. The presence of GM3 in significant proportion was also noticeable in this tissue. This might be due in part to the fact that the retina, despite being a nervous tissue, is largely irrigated by a retinal vascular network, in which GM3 is the major GG. Indeed, GM3 was the most abundant among plasma GGs since it represented 70% of the total nmoles of GGs detected. The plasma also contained significant amounts of GM1 and GD3 but circulating GGs were much less diverse than in the other tissues studied. GM3 was the major GG in the RPE/choroid as well (81% of the total nmoles of GGs detected), which is mainly composed of blood vessels and capillaries. GM1, GD3, GD1a, GD1b and GT1b were also present, in much lower proportions, in this tissue. The GG pattern of the ciliary body was very similar to the RPE/choroid except for the abundance of GD3 (33% of the total nmoles of GGs detected). The GG pattern of the brain was very different from the other tissues with the absence of GM3 and the abundance of tetraosylGG: GM1, GD1a, GD1b and GT1b which represented respectively 23%, 15%, 35% and 26% of the total nmoles of GGs detected. Contrary to the retina, GD3 was a minor class in the brain. The optic nerve exhibited a GG pattern in between the retina and the brain with the presence of hematosides (GM3 and GD3) in addition to an abundance of tetraosylGGs. One unidentified band running just above GD1b was observed in the retina and brain, as well as in the optic nerve and to a lower extent in the ciliary body. Other bands running above GM3 were also detected in the different tissues analyzed. One of them was very prominent in the optic nerve and also present in brain extracts. These less polar GGs likely have an oligosaccharide chain shorter than GM3, the simplest GG of the ganglio-series, and thus belong to another series of GGs.

### Ganglioside classes and their ceramide composition determined by LC/MS

**Fig 3:**
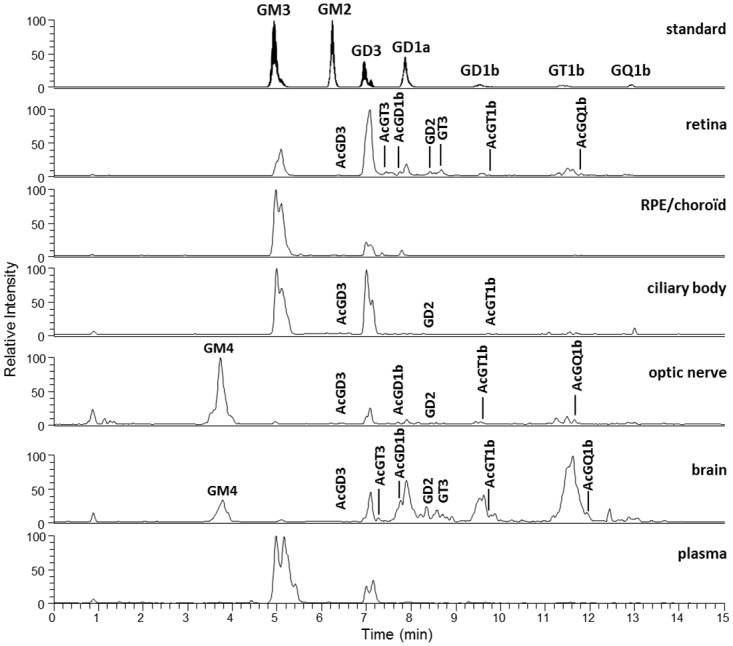
Precursor ion chromatograms of the standard mixture, retina and other ocular tissues, brain and plasma. A representative sample of each tissue was obtained by pooling an aliquot of every GG extract samples (4–7) for each tissue type. The QqQ mass spectrometer was operated in the precursor ion scanning of the characteristic fragment of GG, *N*-Acetylneuraminic acid, at *m/z* 290, in the negative ionization mode.

GGs were further analyzed by LC/MS. Prior to detection, a method of liquid chromatography in HILIC conditions was used for an efficient separation of the ganglio-series GG classes. Mass spectrometry then allowed for the detection of every molecular species, representing different ceramide structures, present in each class, based on their exact mass and charge state. The different GG classes (different sugar chains) and the different ceramide types (total number of carbons and unsaturations) could thus be discriminated. First, a precursor ion scanning of the characteristic fragment of GGs, *N*-Acetylneuraminic acid, at *m/z* 290, was performed in the negative mode using the triple quadrupole (QqQ) mass spectrometer. Fig 3 shows representative total ion chromatograms of the different tissues of interest. GGs corresponding to commercial standards were detected, confirming the identification previously made by HPTLC: GM3, GD3, GD1a, GD1b, GT1b and GQ1b. GM1, which does not form multicharged ions and stands over the mass range of the QqQ mass spectrometer, could not be detected with this apparatus. It should be noted that this LC/MS method is not quantitative. Indeed, the intensity of a GG class peak is not directly proportional to its absolute amount. It also depends on the ability of this specific GG to be fragmented and ionized in the apparatus, which varies among the different GG classes.

Additional unknown peaks could be detected in between major GG classes, especially in the retina, optic nerve and brain. These minor and less frequently reported GGs were identified using high-resolution mass spectrometry. They were analyzed in the positive full scan mode using a LTQ-Orbitrap XL mass spectrometer. Mainly, GD2 and GT3 were identified, which likely correspond to the unidentified band observed in HPTLC running between GD1a and GD1b. Moreover, we were able to detect several acetylated forms of GGs (GD3, GD1b, GT1b, GQ1b, and GT3), especially in the nervous tissues (retina, optic nerve and brain). GM1 was also detected and identified with this apparatus. Thanks to the high mass accuracy of the Orbitrap analyses, we identified the peak detected before GM3 in the optic nerve and brain, previously revealed in HPTLC, as GM4, a gala-series GG. It should be noted that its ceramide contained a hydroxylated fatty acid, which was not observed among the ganglio-series GGs. This specificity of GM4 ceramide has already been reported in human brain [24] and shark liver [25]. GM4 is known to be highly enriched in central nervous system oligodendrocytes that explains its detection in the brain, and in myelin that explains its detection in the optic nerve [8]. As can be seen in Fig 3, the retina and brain presented with the highest diversity of GG classes. Acetylated forms were overall more present in neural tissues (retina, brain and optic nerve) even though AcGD3 and AcGT1b could also be detected in the ciliary body. Interestingly, GT3 and its acetylated forms were specific to the retina and brain.

A huge diversity of molecular species was detected among these different classes of ganglio-series GGs as shown in S1 Table giving an exhaustive list. Overall, ceramides ranged from 32 to 44 carbons with one, two or three unsaturations. The acetylated forms of GGs presented a lower number of ceramide types than the non-acetylated forms. The highest diversity was seen in the retina, in which 108 molecular species of GGs in total were detected. Then, 105 molecular species were detected in the ciliary body and optic nerve, followed by the brain and plasma with 94 and 92 molecular species detected, respectively. The less diverse tissue was the RPE/choroid with 73 detected molecular species of GGs in total. Some species were widely distributed among the different GG classes and tissues of interest, while others appear to be rather specific. Ceramides 34:1, 36:1, 38:1, 40:1, 42:2 and 42:1 were present in almost every GG class of every tissue. On the contrary, ceramides 43:1 and 44:2 were often specific to the optic nerve. The retina specifically expressed GT3 and acetylated GT3 molecular species, which were absent from the other tissues studied except for a few species in the brain. It was noticeable that ceramide 42:3, the only species with 3 unsaturations, was systematically absent from the retina and brain while it was present in various GG classes of the other tissues.

### Molecular species profile of the various GG classes

**Fig 4:**
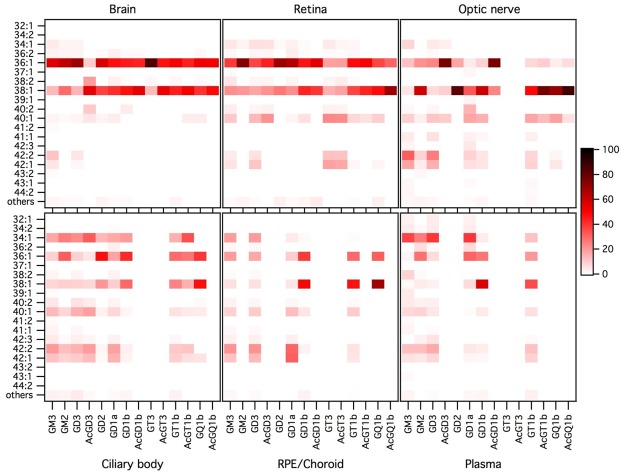
Molecular species profile of the ganglioside classes of the retina and other ocular tissues, brain and plasma. Data (peak areas) were obtained by operating the QqQ mass spectrometer in negative SRM mode. The proportion of each ceramide species was expressed, as percentage, relatively to the sum of all detected species in its specific GG class, every GG class being considered separately. Molecular species accounting for less than 1% were grouped under the category “others”. Results were expressed as mean of 4 to 7 independent samples for each tissue, injected three times. The color intensity of a spot on the graph is proportional to the mean percentage of the ceramide species considered within a GG class.

The proportions of the different molecular species detected, corresponding to different ceramide structures, were calculated for each GG class independently, using the data obtained with the QqQ mass spectrometer operated in the negative SRM mode. The full set of data is given in S2 Table as means and standard deviations of 4–7 independent samples for each tissue, injected three times. In addition, a graphical representation is given in Fig 4.

Among the 19 molecular species of the 14 GG classes and 6 tissues of interest, both tissue and GG class specificities or common features clearly appeared. In an attempt to highlight those features and test their statistical significance, molecular species were grouped according to different structural parameters linked to the chain length or number of unsaturations of the ceramide. The data and the results of their statistical analyses are given in Table 1.

As shown in Fig 4 and Table 1A, ceramides 36:1 and 38:1 were the most abundant species, especially in the retina and brain where they totalized 85 to 100% of all molecular species for most of the GG classes. Retinal GT3 and AcGT3 stood out with a much lower proportion of these two ceramide types (34–38%), while they still accounted for around 60% in GM3, GD3 and AcGD3. Ceramides 36:1 and 38:1 were dominant as well in most optic nerve GG classes. On the contrary, ceramide 34:1 (Table 1B) was absent or very minor in nervous tissues but abundant in the RPE/choroid, ciliary body and especially plasma where its proportion reached almost 40% in some GG classes. Ceramides 40:1, 42:2 and 42:1 (Table 1C) were also very minor in the brain and retina, while quite abundant in the other tissues, especially the optic nerve. Retinal GT3 and AcGT3, and to a lower extent its precursor GD3, made an exception since they contained important levels of these three ceramides (54%, 52% and 32%, respectively).

Regarding the number of unsaturations or the chain length, ceramides with 1 unsaturation and 36 to 41 carbons were by far the most abundant ones. However, polyunsaturated, shorter or longer carbon chain ceramides were also identified. The more complex the oligosaccharide chain, the lower proportion of ceramides with 2 or 3 unsaturations was observed. It appeared that the retina and the brain once again shared some common features. As already mentioned, none of these two tissues contained ceramide species with 3 unsaturations. Moreover, they had significantly higher proportions of monounsaturated ceramides and consecutively lower proportions of ceramides with 2 unsaturations compared to every other tissues of interest (Table 1D and 1E). However, retinal GT3 and AcGT3 differed from the same classes in the brain by the fact that they contained almost 20% of ceramides with 2 unsaturations, 10 times more than brain GT3. The optic nerve, despite being a nervous tissue, often presented similar proportions of polyunsaturated ceramides than the RPE/choroid, ciliary body or plasma. Regarding the ceramide chain length, the retina and brain, along with the optic nerve, exhibited a particularly low proportion of ceramides with 34 carbons or less (Table 1F). On the contrary, the plasma contained important amounts of short ceramides as did the RPE/choroid and the ciliary body. The retina and the brain also contained low proportions of ceramides with 42 carbon-chain length or more (Table 1G). An exception was noted again for retinal GT3 and AcGT3 in which 42 carbon ceramides accounted for about 30% of all molecular species, followed by GD3 (18%).

**Fig 5:**
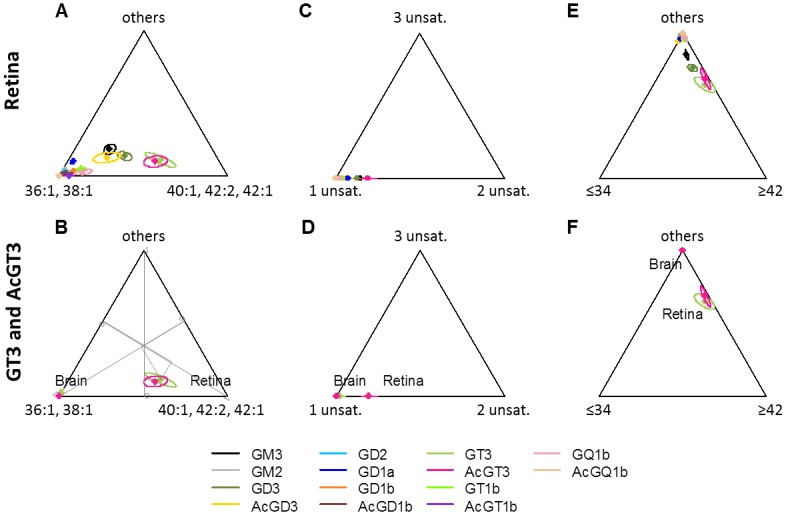
Ceramide proportions in retinal GG classes (A, C, E) and in retinal and brain GT3 and AcGT3 (B, D and F). Means of 4–7 independent samples and 95% confidence ellipses are represented on ternary diagrams for various groups of ceramides: (36:1 + 38:1), (40:1 + 42:2 + 42:1) ceramides and other molecular species (A and B); ceramides with 1, 2 and 3 unsaturations (C and D); ceramides with 34 carbons or less, 42 carbons or more and other molecular species (E and F). Axes from 0 to 1 for the three variables are represented on B and the proportions for retinal GT3 are shown as an example.

Overall, it appeared that retinal GT3 and AcGT3 exhibited specificities in their ceramide composition. This is illustrated in the ternary diagrams shown in Fig 5 representing ceramide profile as average proportions and confidence ellipses of different variables: (36:1 + 38:1), (40:1 + 42:2 + 42:1) ceramides and other molecular species (Fig 5A and 5B); ceramides with 34 carbons or less, 42 carbons or more and other molecular species (Fig 5C and 5D); ceramides with 1, 2 and 3 unsaturations (Fig 5E and 5F). This representation clearly shows that retinal GT3 and AcGT3 have distinctive ceramide profiles, both compared to other retinal GG classes and compared to brain GT3 and AcGT3.

### Ceramide structure characterization by Liquid Chromatography/High Resolution Mass Spectrometry

The ceramide structures (combinations of LCB and FA) were determined after fragmentation of the GG molecular species using the LTQ-Orbitrap mass spectrometer, as detailed previously [1]. The LCB were identified and the associated FA was then deduced based on the exact mass of the molecule. Results obtained for the retina are summarized in Table 2. Results obtained for the other tissues of interest (ciliary body, optic nerve, brain and plasma) are given in S3–S6 Tables. Ceramides of the RPE/choroid could not be characterized because of a lack of sample material. Some GG class molecular species could not be characterized either due to their low amount.

**Table 2:** Ceramide molecular species of the retinal ganglioside classes characterized by HRMS with the LTQ-Orbitrap mass spectrometer.

	GM3	GM2	GD3	AcGD3	GD2	GD1a	GD1b	AcGD1b	GT3	AcGT3	GT1b
**32:1**	N.I.	N.D.	N.D.	N.D.	N.D.	N.D.	N.D.	N.D.	N.D.	N.D.	N.D.
**34:1**	**d16:1/18:0**	N.I.	d16:1/18:0	N.I.	N.I.	N.I.	N.I.	N.I.	N.I.	N.I.	
	**d18:1/16:0**		**d18:1/16:0**								**d18:1/16:0**
**36:2**	d16:1/20:1				N.I.			N.I.	N.I.	N.I.	N.I.
	d17:0/19:2										
	d18:2/18:0		d18:2/18:0								
	**d18:1/18:1**	**d18:1/18:1**	**d18:1/18:1**	**d18:1/18:1**		**d18:1/18:1**	**d18:1/18:1**				
**36:1**	d16:1/20:0										
	d17:0/19:1										
	**d18:1/18:0**	**d18:1/18:0**	**d18:1/18:0**	**d18:1/18:0**	**d18:1/18:0**	**d18:1/18:0**	**d18:1/18:0**	**d18:1/18:0**	**d18:1/18:0**	**d18:1/18:0**	d18:1/18:0
											**d20:1/16:0**
**37:1**	d16:1/21:0	N.D.	d16:1/21:0	N.D.	N.D.	N.D.	N.D.	N.D.	N.D.	N.D.	N.D.
	**d18:1/19:0**		**d18:1/19:0**								
			d20:1/17:0								
**38:2**	d16:1/22:1	N.I.		N.I.	N.I.			N.D.	N.I.	N.I.	N.I.
	d18:2/20:0		d18:2/20:0								
	**d18:1/20:1**		**d18:1/20:1**			**d18:1/20:1**	d18:1/20:1				
	d20:1/18:1						**d20:1/18:1**				
**38:1**	d16:1/22:0							N.I.			N.I.
	d17:0/21:1										
	**d18:1/20:0**	**d18:1/20:0**	**d18:1/20:0**	**d18:1/20:0**	d18:1/20:0	**d18:1/20:0**			**d18:1/20:0**	**d18:1/20:0**	
	d20:1/18:0		d20:1/18:0		**d20:1/18:0**	d20:1/18:0	**d20:1/18:0**				
**39:1**	**d18:1/21:0**	N.I.	N.D.	N.D.	N.D.	N.D.	N.D.	N.D.	N.D.	N.D.	N.D.
**40:2**	d18:2/22:0	N.I.	d18:2/22:0	N.I.	N.D.	N.I.	N.I.	N.D.		N.I.	N.D.
	**d18:1/22:1**		**d18:1/22:1**						**d18:1/22:1**		
**40:1**		N.I.	d17:0/23:1		N.I.	N.I.	N.I.	N.I.			N.I.
	**d18:1/22:0**		**d18:1/22:0**	**d18:1/22:0**					**d18:1/22:0**	**d18:1/22:0**	
**41:1**	**d18:1/23:0**	N.D.	N.D.	N.D.	N.D.	N.D.	N.D.	N.D.	N.D.	N.D.	N.D.
**42:2**	d18:2/24:0	N.D.	d18:2/24:0	N.D.	N.D.	N.I.	N.I.	N.D.			N.I.
	**d18:1/24:1**		**d18:1/24:1**						**d18:1/24:1**	**d18:1/24:1**	
									**d20:1/22:1**		
**42:1**	**d18:1/24:0**	N.D.	**d18:1/24:0**	N.D.	N.D.	N.I.	N.I.	N.D.	d18:1/24:0	**d18:1/24:0**	N.I.
									**d20:1/22:0**		

We were not able to characterize any molecular species in AcGT1b, GQ1b and AcGQ1b. Major molecular species are indicated in bold. N.D.: Non-detected. N.I.: Non-identified.

As expected for mammalian sphingolipids, sphingosine (d18:1) was by far the most widely represented LCB in our tissue samples. Both d16:1 and d20:1, the most common LCB variants reported in the literature were also quite frequently detected. d18:2 was found in the ceramide species with 2 or 3 unsaturations. d17:0 and d17:1 were detected as well, generally as very minor amounts. Those odd species, rarely reported, had nevertheless already been observed in plasma GGs using gas-liquid chromatography [26]. Interestingly, the human retina presented five types of long chain bases: mostly d18:1 but also d16:1, d18:2 and d20:1 quite prominently while d17:0 was frequently present in minor proportions. The presence of d20:1 happened to be specific to nervous tissues (retina, brain and optic nerve). Also, it appeared to be much more represented in b-series GG, especially in the retina where it was almost absent from a-series GG, but also in the brain. This observation was first made in rat brain by Palestini *et al*. [27, 28]. On the contrary, d17:1 was not detected in nervous tissues but only in the plasma and ciliary body. Similarly, d16:1 was mainly found in the plasma and ciliary body, although in minor proportion compared to other LCB, and was practically absent from nervous tissues.

The fatty acids were more diverse than the LCB. They ranged from 14 to 26 carbons and we found no short or medium-chain length. It is noticeable that odd carbon chains such as 15, 17, 19, 21 or 23 carbons occurred. The fatty acids were saturated for the great majority, but also monounsaturated. They rarely presented two unsaturations, only in odd FAs. No fatty acid seemed to be specifically associated to a long chain base, contrary to what had been previously reported in rat brain [28]. In nervous tissues, 18:0 was the most abundant FA, followed by 20:0. The retina and optic nerve also contained significant amounts of 22:0, 24:1, 24:0. Retinal GT3 was characterized by the abundance of 22:1, 22:0 and 24:1 FAs compared to 18:0 and 20:0. The absence of ceramide with 3 unsaturations in the retina and brain resulted in an absence of 24:2 FA from these tissues. The specific representation of ceramides 43:1 and 44:2 in the optic nerve corresponded mainly to the presence of 24:1, 25:0 and 26:1 FAs. The low levels of 34:1 ceramide we observed in nervous tissues was associated with low levels of 16:0 FA. On the contrary, 16:0 was the major FA in the plasma and ciliary body, followed by 24:1. The ciliary body also presented with significant levels of 24:0 and 22:0 FAs.

Those observations resulted in d18:1/18:0 being the most common ceramide type in the retina and brain, followed by d18:1/20:0 and d20:1/18:0. In the optic nerve, d20:1/18:0 was the most prominent, followed by d18:1/18:0. d18:1/16:0 was the major combination in the plasma and ciliary body, followed by d18:1/24:1.

## Discussion

Our comparative analysis of GGs in the retina and other ocular tissues, brain and plasma of aged individuals highlighted tissue specificities in the GG class pattern. The most significant observation we made stands in the specific expression of GD3 and GT3 in the retina. Indeed, the abundance of GD3, as measured by HPTLC and colorimetric revelation, appeared as a distinctive feature of the retina compared to the other nervous tissues, brain and optic nerve, in which GD3 was minor. It is well known that GG levels fluctuate during development of the nervous system and clear correlations have been made between these changes in GG pattern and neurodevelopmental milestones [8]. In the brain, GD3 levels drastically decrease concomitantly with an increase in the levels of more complex gangliotetraosylGGs (GM1, GD1a, GD1b and GT1b) which become prevalent [8]. On the contrary, it has already been shown that GD3 remains a major GG in the adult mammalian retina [16–19], although the retina and the brain derive embryologically from the same origin. GD3’s product via sialyltransferase II, GT3, and its acetylated form, AcGT3, also appeared to be specific to the retina in our study. Apart from a few species detected in the brain, these two GG classes were completely absent from the other tissues we tested. Most of the profiling studies performed on brain GGs of different species (mouse, bovine, porcine, human) using MS do not report on GT3 or AcGT3 [29–33]. However, Ikeda *et al*. [34] detected a few molecular species of GT3 in porcine brain, Vukelic *et al*. in gliosarcoma brain tumor tissue [35] and Zamfir *et al*. in human brain [36]. Regarding the retina, we previously detected several molecular species of GT3 and AcGT3 in rat samples [1]. Literature data regarding GT3 is very poor. GT3 belongs to the c-series GGs frequently referred to as A2B5 antigens. They are expressed in neural stem cells and are markers of glial precursor cells (O-2A progenitor cells) which differentiate into oligodendrocytes and type-2 astrocytes [8]. c-series GGs, especially GT3, were detected in the developing mouse brain using A2B5 antibody. GT3 expression was drastically reduced during development [37]. Besides, the presence of GT3 was reported using another antibody specific to developmentally regulated antigens in chick embryonic retina, optic lobe and cerebrum with a decreased expression in the adult. The authors also detected GT3 in adult human optic nerve, spinal cord and cerebellum but not in non-neural tissues [38]. It is not clear to what cell type this specific expression of GD3, GT3 and AcGT3 we report here in the adult human retina can be attributed. Several observations suggest that b-series GGs, especially GD3, are also markers of neural stem cells and neural precursor cells and that they are implicated in their proliferation in response to growth factor stimulation [39]. This leads to think that the specific expression of GD3 and GT3 in the retina could be due to the persistence of progenitor cells. The specific abundance of GD3 in the retina could more likely be due to retinal specialized cells, the photoreceptors, rods and cones that contain the light-sensitive structures. Indeed, a previous study performed in rat showed that the outer retina, containing the photoreceptors, expressed especially high amounts of GD3 [18]. Interestingly, the authors showed that the photoreceptors expressed low amounts of GGs and exhibited a simplified GG profile compared to the whole retina suggesting specificities in GG expression within the different nervous cell types of the retina. Unravel the presence of GT3 in the retina would now require analyzing GG profile of the different layers of the tissue after microdissection (inner retina vs outer retina) and of isolated cellular populations specific to the retina (photoreceptors, neurons that participate in transmitting signals to the brain such as the retinal ganglion cells or glial cells such as the Müller cells…). Moreover, we cannot rule out that the retinal specificities we observed in the GG profile are not age-related since our samples originated from elderly deceased donors (mean age 87.3 years old).

In addition to tissue specificities in the GG class pattern, our thorough analysis of GGs using LC/MS also revealed specificities in the molecular species, i.e. ceramide type, pattern of tissues and GG classes. While d18:1 appeared as the major LCB in our samples, we report, according to literature data, the presence of d20:1 LCB in the brain. Indeed, this LCB has been commonly described in brain GGs, especially with advanced aged [40]. It is noticeable that nervous system GGs have been described as the only complex sphingolipids to contain significant amounts of d20 LCB, compared to neutral glycosphingolipids, sulfatides and sphingomyelin [40]. From our samples, it appears that d20:1 LCB occurs in other type of nervous tissues as well (retina and optic nerve) while it was absent from non-nervous tissues (RPE/choroid, ciliary body and plasma). A significant proportion of d20:1 LCB was previously described by Holm *et al*. in GGs of bovine retina, especially in GD1a, GD1b and GT1b [41], similarly to our observations. They also reported d20:1 LCB in bovine optic nerve [42]. However, these authors detected low levels of d18:0 and d20:0 LCB. On the contrary, we did not detect any of these two LCB in our samples. It is not clear whether this discrepancy is due to species or age specificities or to differences in the analytical techniques. Besides, we describe the presence of d16:1 LCB, mainly in plasma as previously reported [26], but also in the retina. Serine palmitoyltransferase (SPT) is the enzyme responsible for the synthesis of LCB by condensation of serine and palmitoyl-CoA, its preferred substrate. This leads to d18:1 (sphingosine) being by far the most abundant LCB in sphingolipids, among which GGs, as we report here in our tissues of interest. In addition, SPT can accommodate other fatty acyl-CoAs such as C14:0 or C18:0 giving rise to LCB with 16 and 20 carbons, respectively. However, our analyses revealed small proportions of original LCB with odd (d17:0 and d17:1) and polyunsaturated (d18:2) chain length. Those had not been previously identified in the brain [28, 30, 40], retina or optic nerve [41, 42] but d17:0 and d17:1 had been detected in plasma [26]. Regarding the FA, stearic acid (18:0) is described as the main fatty acid of mammalian nervous system GGs, accounting for over 80% of the total GG FA content [40]. This was the case in our GG extracts from human brain, retina and optic nerve while 16:0 was the main FA in the plasma and ciliary body GGs. However, we also detected a variety of minor FAs in the different tissues we studied. A striking observation was the total absence of long chain polyunsaturated fatty acids (LCPUFAs) in GGs of the retina and brain considering that these tissues contain very high amounts of LCPUFAs, especially docosahexaenoic acid (22:6 DHA): about 20 and 7% of total FAs in the retina and brain cerebrum, respectively [43]. Interestingly, retinal GT3 exhibited specificities in its molecular species composition. Indeed, retinal GT3 and AcGT3 were very similar and differed both from brain GT3 and AcGT3 and from other retinal GG classes by the abundance of ceramides 40:1, 42:2 and 42:1 resulting in specifically high proportions of ceramides with 2 unsaturations and ≥ 42 carbons. These features corresponded to an abundance of 22:0, 22:1 and 24:1 fatty acids.

The tissue specificities in the GG class as well as in the molecular species composition we observed could lead to think that specific GGs might exhibit specific properties relative to the structure and function of the tissue in which they are expressed. However, the biological significance of such individuality remains difficult to decipher. The diversity and biological roles of GGs have long been associated exclusively to differences in the carbohydrate chain protruding toward the extracellular environment. The biological impact of subtle structural variations in the lipid moiety inserted into the outer leaflet of the plasma membrane has just started to be uncovered. The emergence of lipidomic, applied to glycobiology, has helped answer these questions by offering a direct access to the ceramide structures. According to the current raft concept, GGs colocalize with sphingomyelin and cholesterol to form ordered membrane microdomains within a more fluid bilayer mainly composed of phospholipids. These domains with a peculiar lipidic composition also contain numerous signaling proteins and are considered as signaling platforms [44]. Thanks to their unique properties, GGs are able to laterally segregate and thus seem to actively participate in forming and stabilizing membrane microdomains. Actually, GG properties rely both on their hydrophilic oligosaccharide chain and on their hydrophobic ceramide moiety and their redistribution within the plasma membrane might thus be crucial for signaling events occurring at the cell surface [45].

The hydrophilic moiety is believed to participate in determining the clustering of the molecules within the plasma membrane. Since clustering is favored by large differences in the geometrical characteristics in the head group of the complex lipids, GGs that have a much more hydrophilic and larger head group than the neighboring phospholipids are thought to be especially efficient in that matter. This bulky head group requires a wide interfacial area and increasing the volume of the oligosaccharide chain will increase the curvature of the membrane domains. Moreover, the GG head groups are surrounded by a water shell attracted by the hydrophilic character of sugars and by the necessity to avoid repulsion between the negatively charged oligosaccharides. This water stabilizes the GG clustering by organizing a net of hydrogen bonds [46]. As mentioned above, GT3 is a c-series GG, which is characterized by an oligosaccharide chain composed of a sequence of three sialic acids linked to the galactose residue. This unique head group within the GGs detected in our tissue samples must exhibit specific properties in term of geometrical packing and hydrophilic interactions. Indeed, the negatively charged carboxyl group of the three sialic acids would be associated with a particularly large water shell. The dynamic properties of sialylated oligosaccharide chain would also contribute to determine a large interfacial area for this specific GG class. Thus, aggregates of GT3 will likely be strongly excluded from glycerophospholipid areas and cluster to form stable microdomains with a significant membrane curvature [45]. Moreover, the original glycosyl epitope of GT3 must exhibit specific properties in term of interactions with signaling partners. Indeed, the oligosaccharide chain of GGs exposed at the cell surface, whose properties depend on the number, nature and sequence of its residues, is known to be involved in glycosylation-dependent adhesion, recognition and signaling [47].

Regarding the hydrophobic ceramide moiety, the amide group forming a network of hydrogen bonds at the water-lipid interface of the plasma membrane and the double bond of the LCB near this interface are considered to make ceramide a rigid structure and contribute to the decrease in membrane fluidity induced by GGs [46]. Besides, a typical feature of GGs is the high proportion of rigid saturated alkyl chains, which is thought to be crucial for the capabilities of GGs to form membrane domains. Indeed, lipids that contain rigid saturated alkyl chains with high transition temperature are spontaneously excluded from those that contain unsaturated chains with low transition temperature [46]. It is hard to predict what consequences modest differences in the number of unsaturations or chain length of the FA and LCB would have for the cell. Indeed, most of the studies regarding the impact of the ceramide moiety in the partitioning of sphingolipids and GGs in ordered microdomains have been performed using model membranes. For instance, Palestini *et al*. used liposomes composed of a mixture of DEPC (dielaidoylphosphatidylcholine) and DPPC (dipalmitoylphosphatidylcholine) to show that GM1 with 16:0 to 22:0 saturated acyl chains had a marked preference to incorporate into the ordered gel phase, contrary to GM1 with 14:0 acyl chain which preferentially segregated into the disordered liquid phase. Moreover, GM1 with 18:1 and 18:3 unsaturated acyl chains exhibited a much stronger preference for the liquid phase compared to 18:0. The results also showed little effect of d20:1 LCB compared to d18:1 [48]. However, increasing the LCB chain length from 18 to 20 carbons increases the hydrophobic volume and the transition temperature of GG species leading to think that d20:1 LCB must be more segregated and more efficient in reducing membrane fluidity than d18:1 LCB [40]. Sphingosine (d18:1) was by far the main LCB in our GG extracts but d20:1 was also well represented in nervous tissues (brain, retina, optic nerve). According to these observations, their presence should result in increased membrane rigidity compared to the ciliary body which expresses mainly d18:1. In accordance with literature data, 18:0, 20:0 and 16:0 were the major FAs in our tissue samples. Based on the principles mentioned above, the abundance of 18:0 and 20:0 in nervous tissues and the low proportion of 16:0, contrary to the ciliary body, would be associated with more rigid membrane microdomains. The same consequence would result from the prominence of saturated FAs to the detriment of mono or di-unsaturated FA in the retina and the brain. On the contrary, the specific abundance of 22:1, 22:0 and 24:1 in retinal GT3, compared to 18:0 and 20:0 in other GG classes or other nervous tissues would result in a lower rigidity of the domains containing these GG species or favor their localization into more fluid membrane domains. As previously mentioned, LCPUFAs were absent from our GG extracts. Those FA are usually acylated into membrane phospholipids. DHA is particularly present in phosphatidylserine and phosphatidylethanolamine, especially abundant in synaptosomal membranes in the central nervous system and rod outer segments in the retina. Since plasma microdomains are enriched in phosphatidylcholine and depleted in phosphatidylserine [46], DHA is likely excluded from these domains. Apart from the signaling role of LCPUFAs, which can be released from the membrane by phospholipases and converted to eicosanoids, LCPUFAs also exert structural roles within the plasma membrane. The many unsaturations ensure membrane fluidity allowing molecules to move laterally. In the retina, DHA is thought to favor the conformational changes undergone by rhodopsin during the visual process [43]. Obviously, the lipid moieties of GGs with their mainly saturated or monounsaturated alkyl chains undertake an opposite function by favoring membrane rigidity and stabilizing microdomains to create an appropriate microenvironment for specific protein interactions and signaling events.

In conclusion, the present study offers an in-depth characterization of the GG profile of the human retina in comparison with other ocular structures, brain and plasma. It reveals retinal specificities in both GG class and ceramide pattern, especially using a lipidomic approach based on LC/MS. Our work thus paves the way for future research aiming to determine the specific roles of GGs and their ceramide moiety in the retina.

## Supporting Information

S1 Table Molecular species of the ganglioside classes detected with the QqQ and the LTQ-Orbitrap mass spectrometers in the retina and other ocular tissues, brain and plasma. The QqQ mass spectrometer was operated in negative ion mode and the LTQ-Orbitrap mass spectrometer was operated in positive ion mode. MM, theoretical molecular mass. +, detected; -, non-detected.
(PDF)

S2 Table Molecular species profile of the ganglioside classes of the retina and other ocular tissues, brain and plasma. Data were obtained by operating the QqQ mass spectrometer in negative SRM mode. The percentage of each ceramide species was expressed relatively to the sum of all detected species in its specific GG class, every GG class being considered separately. Molecular species accounting for less than 1% were grouped under the category “others”. Results are given as mean and standard deviation of 4 to 7 independent samples for each tissue, injected three times. N.D.: Non-detected (S/N<3); <LOQ: detected but below the limit of quantification (S/N<10).
(PDF)

S3 Table Ceramide molecular species of the ganglioside classes of the ciliary body characterized by HRMS with the LTQ-Orbitrap mass spectrometer. Only the main classes of this tissue (GM3 and GD3) could be characterized. Major molecular species are indicated in bold. N.D.: Non-detected; N.I.: Non-identified.
(PDF)

S4 Table Ceramide molecular species of the ganglioside classes of the optic nerve characterized by HRMS with the LTQ-Orbitrap mass spectrometer. We were not able to characterize any molecular species in AcGD3, GD2, GD1a, AcGT1b, GQ1b and AcGQ1b. Major molecular species are indicated in bold. N.D.: Non-detected; N.I.: Non-identified.
(PDF)

S5 Table Ceramide molecular species of the brain ganglioside classes characterized by HRMS with the LTQ-Orbitrap mass spectrometer. We were not able to characterize any molecular species in the minor classes of GQ1b and AcGQ1b. Major molecular species are indicated in bold. N.D.: Non-detected; N.I.: Non-identified.
(PDF)

S6 Table Ceramide molecular species of the plasma ganglioside classes characterized by HRMS with the LTQ-Orbitrap mass spectrometer. Only the main classes of this tissue (GM3, GM2 and GD3) could be characterized. Major molecular species are indicated in bold. N.D.: Non-detected; N.I.: Non-identified.
(PDF)
