# Supplementary material for: Ganglioside Profiling of the Human Retina: Comparison with Other Ocular Structures, Brain and Plasma Reveals Tissue Specificities
Source: PLoS One. 2016 Dec 20;11(12):e0168794. doi: 10.1371/journal.pone.0168794 (PMC5173345; doi:10.1371/journal.pone.0168794)
Supplement: S1 Table — The QqQ mass spectrometer was operated in negative ion mode and the LTQ-Orbitrap mass spectrometer was operated in positive ion mode. MM, theoretical molecular mass. +, detected; -, non-detected. (PDF) [file pone.0168794.s001.pdf]

S1 Table. Molecular species of the ganglioside classes detected with the QqQ and the LTQ-Orbitrap mass spectrometers in the retina and other ocular tissues, brain and plasma. The QqQ mass spectrometer was operated in negative ion mode and the LTQ-Orbitrap mass spectrometer was operated in positive ion mode. MM, theoretical molecular mass. +, detected; -, non-detected.

Ceramide	MM	Retina	RPE/choroid	Ciliary body	Optic nerve	Brain	Plasma
**GM3**							
**32:2**	1122.666	-	-	-	-	-	+
**32:1**	1124.682	+	+	+	-	+	+
**34:2**	1150.698	-	+	+	-	-	+
**34:1**	1152.713	+	+	+	+	+	+
**36:2**	1178.729	+	+	+	+	+	+
**36:1**	1180.744	+	+	+	+	+	+
**37:1**	1194.760	+	-	-	-	-	-
**38:2**	1206.760	+	+	+	+	+	+
**38:1**	1208.776	+	+	+	+	+	+
**39:1**	1222.791	+	-	+	+	-	+
**40:3**	1232.775	-	+	+	-	-	+
**40:2**	1234.791	+	+	+	+	+	+
**40:1**	1236.807	+	+	+	+	+	+
**41:2**	1248.807	-	-	+	-	-	+
**41:1**	1250.823	+	+	+	+	+	+
**42:3**	1260.807	-	+	+	+	-	-
**42:2**	1262.823	+	+	+	+	+	+
**42:1**	1264.838	+	+	+	+	+	+
**43:2**	1276.838	-	-	-	-	+	+
**43:1**	1278.854	-	-	-	+	-	+
**44:2**	1290.854	-	+	-	+	-	-
**GM2**							
**32:1**	1327.761	-	-	-	-	+	-
**34:2**	1353.777	-	-	-	-	-	+
**34:1**	1355.793	+	+	+	+	+	+
**36:2**	1381.808	+	+	+	+	+	+
**36:1**	1383.824	+	+	+	+	+	+
**38:2**	1409.839	+	+	+	+	+	+
**38:1**	1411.855	+	+	+	+	+	+
**39:1**	1425.871	+	-	-	-	-	-
**40:2**	1437.871	+	+	+	-	-	+
**40:1**	1439.886	+	+	+	+	+	+
**42:3**	1463.886	-	+	+	-	-	+
**42:2**	1465.902	-	+	+	+	+	+
**42:1**	1467.918	-	+	+	+	+	+
**43:2**	1479.917	-	-	-	+	-	-
**43:1**	1481.933	-	-	-	+	-	-
**44:2**	1493.933	-	-	-	+	-	-

**GM1**							
**32:1**	1489.814	-	-	-	-	-	+
**34:2**	1515.830	-	-	-	-	-	+
**34:1**	1517.845	-	+	+	+	+	+
**36:2**	1543.861	-	-	-	-	+	+
**36:1**	1545.877	+	+	+	+	+	+
**38:2**	1571.892	-	-	-	+	+	+
**38:1**	1573.908	+	+	+	+	+	+
**40:2**	1599.924	-	+	-	+	-	+
**40:1**	1601.939	+	+	+	+	+	+
**42:3**	1625.939	-	+	-	+	-	-
**42:2**	1627.955	+	+	+	+	+	-
**42:1**	1629.971	-	-	+	-	+	-
**43:2**	1641.970	-	-	-	+	-	-
**44:2**	1655.986	-	-	-	+	-	-
**GD3**							
**32:2**	1413.761	-	-	-	-	-	+
**32:1**	1415.777	-	+	+	-	-	+
**34:2**	1441.793	-	+	+	-	-	+
**34:1**	1443.809	+	+	+	+	+	+
**36:2**	1469.824	+	+	+	+	+	+
**36:1**	1471.840	+	+	+	+	+	+
**37:1**	1485.855	+	-	-	-	-	-
**38:2**	1497.856	+	+	+	+	+	+
**38:1**	1499.871	+	+	+	+	+	+
**39:1**	1513.887	-	-	+	-	-	-
**40:2**	1525.887	+	+	+	+	+	+
**40:1**	1527.902	+	+	+	+	+	+
**41:2**	1539.902	-	-	+	-	-	-
**41:1**	1541.918	-	+	+	+	-	+
**42:3**	1551.902	-	+	+	-	-	+
**42:2**	1553.918	+	+	+	+	+	+
**42:1**	1555.934	+	+	+	+	+	+
**43:1**	1569.949	-	-	-	+	-	-
**44:2**	1581.949	-	-	-	+	-	-
**AcGD3**							
**34:1**	1485.819	+	-	+	+	-	-
**36:2**	1511.835	+	-	-	+	-	-
**36:1**	1513.850	+	-	+	+	+	-
**38:2**	1539.866	+	-	+	-	+	-
**38:1**	1541.882	+	-	+	+	+	-
**40:2**	1567.897	+	-	+	-	+	-
**40:1**	1569.913	+	-	+	+	+	-
**41:1**	1583.929	-	-	+	-	-	-
**42:3**	1593.913	-	-	+	-	-	-
**42:2**	1595.929	-	-	+	-	-	-
**42:1**	1597.944	-	-	+	-	-	-

**GD2**							
**34:1**	1646.888	+	-	+	+	+	+
**36:2**	1672.904	+	-	-	-	+	-
**36:1**	1674.919	+	-	+	+	+	+
**38:2**	1700.935	+	-	-	-	+	-
**38:1**	1702.951	+	-	+	+	+	+
**40:2**	1728.966	-	-	-	-	-	-
**40:1**	1730.982	+	-	+	-	+	-
**42:2**	1756.998	-	-	+	-	+	-
**42:1**	1759.006	-	-	+	-	-	-
**GD1a**							
**32:1**	1780.909	-	-	+	-	-	+
**34:2**	1806.925	-	-	+	-	-	+
**34:1**	1808.941	+	+	+	-	+	+
**36:2**	1834.956	+	+	+	+	+	+
**36:1**	1836.972	+	+	+	+	+	+
**38:2**	1862.988	+	+	+	+	+	+
**38:1**	1865.003	+	+	+	+	+	+
**39:1**	1879.019	-	-	-	+	-	-
**40:2**	1891.019	+	+	+	+	-	+
**40:1**	1893.035	+	+	+	+	+	+
**41:2**	1905.034	-	-	+	-	-	-
**41:1**	1907.050	-	-	+	+	-	+
**42:3**	1917.034	-	+	+	+	-	-
**42:2**	1919.050	+	+	+	+	+	+
**42:1**	1921.066	+	+	+	+	+	+
**43:1**	1935.082	-	-	-	+	-	-
**44:2**	1947.082	-	-	-	+	-	-
**GD1b**							
**34:1**	1808.941	+	+	+	+	-	+
**36:2**	1834.956	+	-	-	+	+	-
**36:1**	1836.972	+	+	+	+	+	+
**38:2**	1862.988	+	-	-	+	+	-
**38:1**	1865.003	+	+	+	+	+	+
**40:2**	1891.019	+	+	+	-	-	+
**40:1**	1893.035	+	+	+	+	+	+
**41:1**	1907.050	-	-	+	+	-	+
**42:3**	1917.034	-	-	-	+	-	-
**42:2**	1919.050	+	+	+	+	+	+
**42:1**	1921.066	+	+	+	+	+	+
**43:1**	1935.082	-	-	-	+	-	-
**44:2**	1947.082	-	-	-	+	-	-
**AcGD1b**							
**36:2**	1876.967	+	-	-	-	+	-
**36:1**	1878.983	+	-	-	+	+	-
**38:1**	1907.014	+	-	-	+	+	-
**40:1**	1935.046	+	-	-	-	+	-

**GT3**							
**34:1**	1734.904	+	-	-	-	-	-
**36:2**	1760.920	+	-	-	-	+	-
**36:1**	1762.935	+	-	-	-	+	-
**38:2**	1788.951	+	-	-	-	-	-
**38:1**	1790.967	+	-	-	-	+	-
**40:2**	1816.982	+	-	-	-	-	-
**40:1**	1818.998	+	-	-	-	-	-
**42:2**	1845.014	+	-	-	-	-	-
**42:1**	1847.029	+	-	-	-	-	-
**AcGT3**							
**34:1**	1776.915	+	-	-	-	-	-
**36:2**	1802.930	+	-	-	-	-	-
**36:1**	1804.946	+	-	-	-	+	-
**38:2**	1830.962	+	-	-	-	+	-
**38:1**	1832.977	+	-	-	-	+	-
**40:2**	1858.993	+	-	-	-	-	-
**40:1**	1861.008	+	-	-	-	-	-
**42:2**	1887.024	+	-	-	-	-	-
**42:1**	1889.040	+	-	-	-	-	-
**GT1b**							
**32:1**	2072.005	-	-	-	-	-	+
**34:2**	2098.021	-	-	-	-	-	+
**34:1**	2100.036	+	+	+	+	+	+
**36:2**	2126.052	+	-	+	-	-	+
**36:1**	2128.067	+	+	+	+	+	+
**38:2**	2154.083	+	-	+	-	+	+
**38:1**	2156.099	+	+	+	+	+	+
**39:1**	2170.114	-	-	-	+	-	-
**40:2**	2182.114	-	+	+	+	-	+
**40:1**	2184.130	+	+	+	+	+	+
**41:1**	2198.146	-	-	+	+	-	+
**42:3**	2208.130	-	+	+	-	-	+
**42:2**	2210.146	+	+	+	+	-	+
**42:1**	2212.161	+	+	+	+	+	+
**43:1**	2226.177	-	+	-	+	-	-
**44:2**	2238.177	-	-	-	+	-	-
**AcGT1b**							
**34:1**	2141.047	-	-	+	-	-	-
**36:2**	2168.062	-	-	-	-	+	-
**36:1**	2170.078	+	-	+	+	+	+
**38:1**	2198.109	+	-	+	+	+	+
**40:1**	2226.141	+	-	+	+	+	-
**42:2**	2252.156	-	-	+	-	-	-
**42:1**	2254.172	-	-	+	-	-	-
**GQ1b**							
**34:1**	2391.132	+	-	-	-	-	-

**36:2**	2417.147	**+**	-	-	-	**+**	-
**36:1**	2419.163	**+**	**+**	**+**	**+**	**+**	**+**
**38:2**	2445.179	**+**	-	-	-	**+**	-
**38:1**	2447.194	**+**	**+**	**+**	**+**	**+**	**+**
**40:2**	2473.210	**+**	-	**+**	**+**	-	-
**40:1**	2475.226	**+**	-	**+**	**+**	**+**	-
**42:2**	2501.241	**+**	-	-	**+**	-	-
**42:1**	2503.257	**+**	-	**+**	**+**	-	-
**AcGQ1b**							
**36:1**	2461.174	**+**	-	-	**+**	**+**	-
**38:1**	2489.205	**+**	-	-	**+**	**+**	-
**40:1**	2517.237	-	-	-	**+**	-	-
