# Supplementary material for: Ganglioside Profiling of the Human Retina: Comparison with Other Ocular Structures, Brain and Plasma Reveals Tissue Specificities
Source: PLoS One. 2016 Dec 20;11(12):e0168794. doi: 10.1371/journal.pone.0168794 (PMC5173345; doi:10.1371/journal.pone.0168794)
Supplement: S2 Table — Data were obtained by operating the QqQ mass spectrometer in negative SRM mode. The percentage of each ceramide species was expressed relatively to the sum of all detected species in its specific GG class, every GG class being considered separately. Molecular species accounting for less than 1% were grouped under the category “others”. Results are given as mean and standard deviation of 4 to 7 independent samples for each tissue, injected three times. N.D.: Non-detected (S/N<3); <LOQ: detected but below the limit of quantification (S/N<10). (PDF) [file pone.0168794.s002.pdf]

S2 Table. Molecular species profile of the ganglioside classes of the retina and other ocular tissues, brain and plasma. Data were obtained by operating the QqQ mass spectrometer in negative SRM mode. The percentage of each ceramide species was expressed relatively to the sum of all detected species in its specific GG class, every GG class being considered separately. Molecular species accounting for less than 1% were grouped under the category "others". Results are given as mean and standard deviation of 4 to 7 independent samples for each tissue, injected three times. N.D.: Non-detected (S/N<3); <LOQ: detected but below the limit of quantification (S/N<10).

**Retina**	GM3		GM2		GD3		AcGD3		GD2		GD1a		GD1b		AcGD1b		GT3		AcGT3		GT1b		AcGT1b		GQ1b		AcGQ1b	
Ceramide	Mean (%)	Std. dev.	Mean (%)	Std. dev.	Mean (%)	Std. dev.	Mean (%)	Std. dev.	Mean (%)	Std. dev.	Mean (%)	Std. dev.	Mean (%)	Std. dev.	Mean (%)	Std. dev.	Mean (%)	Std. dev.	Mean (%)	Std. dev.	Mean (%)	Std. dev.	Mean (%)	Std. dev.	Mean (%)	Std. dev.	Mean (%)	Std. dev.
34:1	4.96	0.58	2.52	0.74	6.36	1.29	5.92	0.47	1.33	0.52	3.14	0.35	1.04	0.27	<LOQ		4.45	2.44	2.05	0.78	1.56	0.22	N.D.		<1%		N.D.	
36:2	1.59	0.15	<1%		2.26	0.35	1.96	0.43	<1%		3.69	0.40	0.94	0.36	<1%		1.47	0.35	1.13	0.31	1.73	0.27	N.D.		<1%		N.D.	
36:1	38.74	2.69	73.92	2.08	36.43	2.55	43.75	5.38	68.89	1.53	64.68	2.24	46.38	3.47	55.72	6.08	18.81	2.49	19.25	1.95	47.92	3.76	50.67	5.63	36.72	6.57	30.79	4.39
37:1	1.17	0.12	N.D.		<1%		N.D.		N.D.		N.D.		N.D.		N.D.		N.D.		N.D.		N.D.		N.D.		N.D.		N.D.	
38:2	5.43	1.26	<1%		2.19	0.50	1.98	0.53	<1%		2.52	0.24	<1%		N.D.		2.14	0.48	3.81	1.55	1.23	0.20	N.D.		<1%		N.D.	
38:1	22.21	1.13	20.90	1.32	17.87	0.72	21.76	1.39	26.65	1.11	22.32	1.84	43.43	2.63	39.66	5.28	15.66	1.31	18.69	3.72	37.05	2.50	43.68	4.81	48.01	4.43	69.21	4.39
40:2	3.73	0.86	<LOQ		2.06	0.27	2.87	0.86	N.D.		<1%		<1%		N.D.		3.04	0.64	2.62	0.48	<LOQ		N.D.		<1%		N.D.	
40:1	11.00	0.96	<1%		14.20	1.13	21.78	3.81	<1%		2.38	0.45	6.30	0.53	<1%		23.56	2.04	22.78	2.61	7.87	0.96	6.14	1.46	9.71	1.81	N.D.	
42:2	4.16	0.69	N.D.		6.06	0.92	N.D.		N.D.		<1%		<1%		N.D.		12.28	2.22	11.85	1.49	<1%		N.D.		<1%		N.D.	
42:1	5.53	0.65	N.D.		11.64	1.20	N.D.		N.D.		<1%		<1%		N.D.		18.63	2.62	17.42	3.04	1.77	0.31	N.D.		2.24	0.75	N.D.	
others	1.49	0.21	2.66	0.75	0.93	0.14			3.20	1.06	1.27	0.47	2.09	0.60	4.61	2.10					0.90	0.24			3.32	1.19		

**RPE/Choroid**	GM3		GD3		GD1a		GD1b		GT1b		GQ1b	
Ceramide	Mean (%)	Std. dev.	Mean (%)	Std. dev.	Mean (%)	Std. dev.	Mean (%)	Std. dev.	Mean (%)	Std. dev.	Mean (%)	Std. dev.
34:1	19.25	2.40	19.59	0.61	4.30	1.00	1.21	0.35	1.02	0.41	N.D.	
36:2	1.55	0.30	1.92	0.79	<1%		N.D.		N.D.		N.D.	
36:1	19.57	3.29	17.52	4.70	9.90	1.94	38.73	13.75	29.35	8.51	31.65	7.01
38:2	1.06	0.13	1.38	0.35	<1%		N.D.		N.D.		N.D.	
38:1	6.50	0.54	6.24	1.25	5.26	2.01	49.20	12.82	46.79	2.98	68.35	7.01
40:2	2.80	0.12	2.29	0.14	1.77	0.83	<1%		1.17	1.04	N.D.	
40:1	9.35	0.52	10.40	1.08	13.24	1.02	5.22	1.53	10.18	2.42	N.D.	
41:1	1.56	0.18	1.23	0.18	N.D.		N.D.		N.D.		N.D.	
42:3	3.43	0.37	3.21	0.52	3.22	0.28	N.D.		<1%		N.D.	
42:2	21.36	2.77	21.56	3.15	30.29	1.47	3.13	2.38	5.70	2.71	N.D.	
42:1	11.83	1.67	13.90	2.06	31.27	2.18	<1%		5.44	3.00	N.D.	
others	1.73	0.18	1.34	0.55	0.38	0.08	2.77	2.82	0.53	0.20		

**Ciliary body**	GM3		GM2		GD3		AcGD3		GD2		GD1a		GD1b		GT1b		AcGT1b		GQ1b	
Ceramide	Mean (%)	Std. dev.	Mean (%)	Std. dev.	Mean (%)	Std. dev.	Mean (%)	Std. dev.	Mean (%)	Std. dev.	Mean (%)	Std. dev.	Mean (%)	Std. dev.	Mean (%)	Std. dev.	Mean (%)	Std. dev.	Mean (%)	Std. dev.
32:1	<1%		N.D.		<1%		N.D.		N.D.		<1%		<LOQ		N.D.		N.D.		N.D.	
34:2	<1%		N.D.		<1%		N.D.		N.D.		<1%		<1%		N.D.		N.D.		N.D.	
34:1	17.48	0.47	25.54	13.66	23.01	3.84	32.09	10.26	14.14	4.78	16.99	3.52	19.75	12.42	15.88	8.38	34.71	16.00	N.D.	
36:2	<1%		<1%		<1%		N.D.		N.D.		3.42	0.63	<1%		<1%		N.D.		N.D.	
36:1	9.02	0.46	31.72	13.10	9.13	0.89	5.19	1.33	47.52	8.77	18.03	3.00	41.94	5.80	29.14	4.57	27.17	2.75	39.64	4.46
38:2	2.36	0.56	<1%		2.57	0.85	<1%		N.D.		2.51	1.60	<1%		<1%		N.D.		N.D.	
38:1	8.23	0.34	9.52	4.09	9.32	0.56	8.07	0.14	20.77	10.77	9.80	3.21	29.86	8.15	24.93	5.91	15.84	5.47	47.40	6.93
39:1	1.13	0.20	N.D.		<1%		N.D.		N.D.		N.D.		N.D.		N.D.		N.D.		N.D.	
40:2	4.88	0.57	<1%		5.56	0.82	3.59	1.06	N.D.		2.60	0.36	<1%		<1%		N.D.		1.19	0.27
40:1	13.75	0.66	8.41	1.20	15.67	1.45	18.78	3.53	2.91	0.39	7.93	0.51	2.11	0.98	6.17	1.96	4.38	1.08	4.19	1.25
41:2	1.01	0.09	N.D.		<1%		N.D.		N.D.		<1%		<1%		N.D.		N.D.		N.D.	
41:1	2.43	0.15	N.D.		1.61	0.40	<1%		N.D.		<1%		<1%		<1%		N.D.		N.D.	
42:3	4.40	0.41	1.29	0.32	3.41	0.41	2.19	0.54	N.D.		3.09	0.98	<1%		1.82	0.66	N.D.		N.D.	
42:2	20.04	1.40	13.10	2.91	15.41	1.37	19.54	1.69	2.30	1.05	18.59	4.93	3.46	1.31	10.95	1.93	11.50	3.57	N.D.	
42:1	12.39	0.66	8.84	1.90	10.19	1.22	13.44	3.04	1.25	0.86	15.52	3.52	2.51	1.70	9.41	1.77	6.59	2.30	5.75	2.01
others	2.87	0.18	2.49	1.08	4.12	0.56	1.50	1.21			1.56	0.81	2.34	0.92	2.89	1.53				

**Optic Nerve**	GM3		GM2		GD3		AcGD3		GD2		GD1a		GD1b		AcGD1b		GT1b		AcGT1b		GQ1b		AcGQ1b	
Ceramide	Mean (%)	Std. dev.	Mean (%)	Std. dev.	Mean (%)	Std. dev.	Mean (%)	Std. dev.	Mean (%)	Std. dev.	Mean (%)	Std. dev.	Mean (%)	Std. dev.	Mean (%)	Std. dev.	Mean (%)	Std. dev.	Mean (%)	Std. dev.	Mean (%)	Std. dev.	Mean (%)	Std. dev.
34:1	8.28	1.35	<1%		4.67	1.22	3.46	1.15	<1%		N.D.		<1%		N.D.		<1%		N.D.		N.D.		N.D.	
36:2	<1%		<1%		1.48	0.61	<1%		N.D.		<1%		<1%		N.D.		N.D.		N.D.		N.D.		N.D.	
36:1	12.67	1.86	22.15	1.65	23.94	3.94	75.93	6.76	17.77	3.57	4.63	2.20	9.23	0.48	76.03	2.56	7.60	0.86	9.79	1.31	3.78	0.60	6.66	0.17
38:2	<1%		<1%		<1%		N.D.		N.D.		1.90	1.04	<1%		N.D.		N.D.		N.D.		N.D.		N.D.	
38:1	5.34	0.37	58.84	6.20	7.07	1.30	9.43	5.00	81.87	3.66	32.93	6.33	60.12	5.48	23.97	2.56	48.92	4.54	77.14	1.50	69.93	3.57	87.25	4.13
39:1	<1%		N.D.		N.D.		N.D.		N.D.		2.47	1.19	<1%		N.D.		<1%		N.D.		N.D.		N.D.	
40:2	1.67	0.31	N.D.		2.05	0.26	N.D.		N.D.		15.03	3.51	<1%		N.D.		<1%		N.D.		<1%		N.D.	
40:1	9.69	1.47	3.33	0.85	11.36	1.63	10.45	2.55	N.D.		17.90	2.39	15.75	1.37	N.D.		17.94	1.12	13.54	2.32	18.90	1.52	6.07	2.35
41:1	4.46	1.03	N.D.		6.03	0.83	N.D.		N.D.		2.82	0.11	1.95	0.26	N.D.		3.35	0.57	N.D.		N.D.		N.D.	
42:3	2.20	0.48	N.D.		N.D.		N.D.		N.D.		4.92	3.01	<1%		N.D.		N.D.		N.D.		N.D.		N.D.	
42:2	32.01	2.51	8.64	3.13	25.70	0.96	N.D.		N.D.		9.33	3.89	5.39	1.72	N.D.		8.35	1.27	N.D.		2.27	0.52	N.D.	
42:1	18.03	0.97	3.91	1.65	15.75	1.78	N.D.		N.D.		6.30	2.45	5.42	1.52	N.D.		9.10	1.28	N.D.		4.84	1.51	N.D.	
43:2	N.D.		1.57	0.60	N.D.		N.D.		N.D.		N.D.		N.D.		N.D.		N.D.		N.D.		N.D.		N.D.	
43:1	1.61	0.36	<1%		<1%		N.D.		N.D.		1.43	0.54	<1%		N.D.		1.81	0.19	N.D.		N.D.		N.D.	
44:2	2.18	0.52	<1%		<1%		N.D.		N.D.		<1%		<1%		N.D.		1.34	0.23	N.D.		N.D.		N.D.	
others	1.94	0.34	2.49	1.03	1.94	0.32	0.73	0.88	0.37	0.23	1.00	0.36	2.13	0.52			1.58	0.12			0.29	0.09		

**Brain**	GM3		GM2		GD3		AcGD3		GD2		GD1a		GD1b		AcGD1b		GT3		AcGT3		GT1b		AcGT1b		GQ1b		AcGQ1b	
Ceramide	Mean (%)	Std. dev.	Mean (%)	Std. dev.	Mean (%)	Std. dev.	Mean (%)	Std. dev.	Mean (%)	Std. dev.	Mean (%)	Std. dev.	Mean (%)	Std. dev.	Mean (%)	Std. dev.	Mean (%)	Std. dev.	Mean (%)	Std. dev.	Mean (%)	Std. dev.	Mean (%)	Std. dev.	Mean (%)	Std. dev.	Mean (%)	Std. dev.
32:1	<1%		<1%		N.D.		N.D.		N.D.		N.D.		N.D.		N.D.		N.D.		N.D.		N.D.		N.D.		N.D.		N.D.	
34:1	4.32	1.12	2.72	0.85	2.38	0.35	N.D.		<1%		<1%		<1%		N.D.		N.D.		N.D.		<1%		N.D.		N.D.		N.D.	
36:2	3.04	0.91	2.43	0.43	2.58	0.47	N.D.		<1%	0.24	3.24	0.80	1.45	0.23	1.41	0.71	N.D.		N.D.		N.D.		<1%		1.91	0.41	N.D.	
36:1	56.00	5.24	62.13	7.54	69.35	4.10	9.97	3.82	57.32	8.03	46.92	5.85	44.66	6.45	43.58	5.34	84.68	4.78	46.72	5.25	51.51	6.52	43.79	6.20	52.05	6.35	51.25	7.52
38:2	<1%		<1%		<1%		19.44	1.79	<1%		3.23	0.67	1.14	0.17	N.D.		1.80	1.03	<LOQ		1.70	0.10	N.D.		1.43	0.26	N.D.	
38:1	13.66	3.99	29.08	7.90	14.57	4.45	54.55	5.43	37.38	7.67	41.51	6.23	47.14	6.34	55.02	5.97	13.53	5.13	53.28	5.25	44.40	6.76	51.82	5.84	40.54	6.23	48.75	7.52
40:2	<1%		<1%		<1%		10.72	1.30	N.D.		N.D.		N.D.		N.D.		N.D.		N.D.		N.D.		N.D.		N.D.		N.D.	
40:1	2.59	1.07	1.23	0.51	2.47	0.35	5.31	1.32	2.57	0.76	3.80	0.42	4.24	0.48	<LOQ		N.D.		N.D.		<1%		3.56	0.60	4.11	0.51	N.D.	
41:1	1.00	0.38	N.D.		N.D.		N.D.		N.D.		N.D.		N.D.		N.D.		N.D.		N.D.		N.D.		N.D.		N.D.		N.D.	
42:2	12.12	1.62	<1%		5.17	0.53	N.D.		<1%		<1%		<1%		N.D.		N.D.		N.D.		N.D.		N.D.		N.D.		N.D.	
42:1	5.16	0.91	<1%		1.94	0.14	N.D.		N.D.		<1%		<1%		N.D.		N.D.		N.D.		<1%		N.D.		N.D.		N.D.	
43:2	<1%		N.D.		N.D.		N.D.		N.D.		N.D.		N.D.		N.D.		N.D.		N.D.		N.D.		N.D.		N.D.		N.D.	
others	2.11	0.33	1.29	0.39	1.54	0.23			2.73	0.47	1.30	0.21	1.37	0.24							2.39	0.33	0.83	0.21				

**Plasma**	GM3		GM2		GD3		GD1a		GD1b		GT1b	
Ceramide	Mean (%)	Std. dev.	Mean (%)	Std. dev.	Mean (%)	Std. dev.	Mean (%)	Std. dev.	Mean (%)	Std. dev.	Mean (%)	Std. dev.
32:2	<1%		N.D.		<1%		N.D.		N.D.		N.D.	
32:1	2.87	0.40	N.D.		4.19	0.98	3.10	1.74	N.D.		<1%	
34:2	4.02	0.94	1.37	0.61	4.04	1.20	3.81	0.85	<1%		<1%	
34:1	36.92	5.52	25.48	5.74	39.34	7.50	39.49	7.32	4.21	4.19	12.15	3.69
36:2	2.39	0.58	6.86	2.72	1.48	0.39	7.32	1.77	<1%		<1%	
36:1	9.26	1.52	27.86	10.76	7.12	3.48	30.63	7.54	27.09	14.95	20.53	9.20
38:2	1.33	0.29	1.18	0.78	<1%		<1%		<1%		<1%	
38:1	4.57	0.62	4.16	1.50	2.89	1.14	7.05	4.59	56.93	14.28	35.54	10.30
39:1	<1%		N.D.		N.D.		N.D.		N.D.		N.D.	
40:2	<1%		3.04	1.79	3.47	1.02	<1%		<1%		<1%	
40:1	4.30	0.52	8.65	3.11	5.07	1.27	1.10	0.55	4.49	1.61	6.39	1.60
41:2	7.78	0.89	N.D.		N.D.		N.D.		N.D.		N.D.	
41:1	1.21	0.33	N.D.		1.15	0.30	<1%		<1%		<1%	
42:3	2.43	0.67	3.03	1.12	5.73	1.39	N.D.		N.D.		1.46	1.24
42:2	12.79	1.84	13.25	3.48	17.08	4.36	4.93	1.07	3.53	3.20	12.37	5.19
42:1	6.22	0.79	4.76	1.91	7.65	1.65	2.07	1.10	1.65	1.32	9.31	4.48
43:2	<1%		N.D.		N.D.		N.D.		N.D.		N.D.	
43:1	1.42	0.50	N.D.		N.D.		N.D.		N.D.		N.D.	
others	2.68	0.57			0.88	0.29	1.59	0.49	2.08	0.98	3.23	1.57
