# Supplementary material for: Ganglioside Profiling of the Human Retina: Comparison with Other Ocular Structures, Brain and Plasma Reveals Tissue Specificities
Source: PLoS One. 2016 Dec 20;11(12):e0168794. doi: 10.1371/journal.pone.0168794 (PMC5173345; doi:10.1371/journal.pone.0168794)
Supplement: S3 Table — Only the main classes of this tissue (GM3 and GD3) could be characterized. Major molecular species are indicated in bold. N.D.: Non-detected; N.I.: Non-identified. (PDF) [file pone.0168794.s003.pdf]

S3 Table. Ceramide molecular species of the ganglioside classes of the ciliary body characterized by HRMS with the LTQ-Orbitrap mass spectrometer. Only the main classes of this tissue (GM3 and GD3) could be characterized. Major molecular species are indicated in bold. N.D.: Non-detected. N.I.: Non-identified.

	GM3	GD3
**32:1**	N.I.	**d18:1/14:0**
**34:2**	**d18:1/16:1**	**d18:1/16:1**
**34:1**	**d18:1/16:0**	**d18:1/16:0**
**36:2**	d16:1/20:1	
	d18:2/18:0	
	**d18:1/18:1**	**d18:1/18:1**
**36:1**	d16:1/20:0	d16:1/20:0
	**d18:1/18:0**	**d18:1/18:0**
**38:2**	d18:2/20:0	d18:2/20:0
	**d18:1/20:1**	**d18:1/20:1**
**38:1**	**d18:1/20:0**	**d18:1/20:0**
**39:1**	d17:1/22:0	
	**d18:1/21:0**	**d18:1/21:0**
**40:3**	d18:2/22:1	N.D.
	**d18:1/22:2**	
**40:2**	d18:2/22:0	d18:2/22:0
	**d18:1/22:1**	**d18:1/22:1**
**40:1**	**d18:1/22:0**	**d18:1/22:0**
**41:2**	**d18:1/23:1**	**d18:1/23:1**
**41:1**	d17:1/24:0	
	**d18:1/23:0**	**d18:1/23:0**
**42:3**	d17:0/25:3	
	d18:2/24:1	d18:2/24:1
	**d18:1/24:2**	**d18:1/24:2**
**42:2**	d17:0/25:2	
	d18:2/24:0	d18:2/24:0
	**d18:1/24:1**	**d18:1/24:1**
**42:1**	**d18:1/24:0**	**d18:1/24:0**
