# Supplementary material for: Ganglioside Profiling of the Human Retina: Comparison with Other Ocular Structures, Brain and Plasma Reveals Tissue Specificities
Source: PLoS One. 2016 Dec 20;11(12):e0168794. doi: 10.1371/journal.pone.0168794 (PMC5173345; doi:10.1371/journal.pone.0168794)
Supplement: S4 Table — We were not able to characterize any molecular species in AcGD3, GD2, GD1a, AcGT1b, GQ1b and AcGQ1b. Major molecular species are indicated in bold. N.D.: Non-detected; N.I.: Non-identified. (PDF) [file pone.0168794.s004.pdf]

S4 Table. Ceramide molecular species of the ganglioside classes of the optic nerve characterized by HRMS with the LTQ-Orbitrap mass spectrometer. We were not able to characterize any molecular species in AcGD3, GD2, GD1a, AcGT1b, GQ1b and AcGQ1b. Major molecular species are indicated in bold. N.D.: Non-detected. N.I.: Non-identified.

	**GM3**	**GM2**	**GD3**	**GD1b**	**GT1b**
**34:1**	**d18:1/16:0**	N.I.	**d18:1/16:0**	N.I.	N.I.
**36:2**	**d18:1/18:1**	N.I.	**d18:1/18:1**	**d18:1/18:1**	N.D.
**36:1**	**d18:1/18:0**	N.I.	**d18:1/18:0**	**d18:1/18:0**	N.I.
**38:2**	d18:2/20:0	N.I.			N.D.
	**d18:1/20:1**		**d18:1/20:1**		
	**d20:1/18:1**		d20:1/18:1	**d20:1/18:1**	
**38:1**	**d18:1/20:0**	N.I.	**d18:1/20:0**		N.I.
	**d20:1/18:0**		d20:1/18:0	**d20:1/18:0**	
**39:1**	**d18:1/21:0**	N.D.	N.D.	N.D.	N.I.
	d20:1/19:0				
**40:2**	**d18:1/22:1**	N.D.	**d18:1/22:1**	N.D.	N.I.
**40:1**	**d18:1/22:0**	N.I.	**d18:1/22:0**		**d18:1/22:0**
				**d20:1/20:0**	
**41:1**	**d18:1/23:0**	N.D.	**d18:1/23:0**	**d18:1/23:0**	N.I.
	d20:1/21:0				
**42:3**	**d18:1/24:2**	N.D.	N.D.	**d18:1/24:2**	N.D.
**42:2**	**d18:1/24:1**	**d18:1/24:1**	**d18:1/24:1**	**d18:1/24:1**	N.I.
			d20:1/22:1		
**42:1**	**d18:1/24:0**	**d18:1/24:0**	**d18:1/24:0**	**d18:1/24:0**	N.I.
			d20:1/22:0		
**43:2**	N.D.	**d18:1/25:1**	N.D.	N.D.	N.D.
**43:1**	**d18:1/25:0**	N.I.	**d18:1/25:0**	N.I.	N.I.
	d20:1/23:0				
**44:2**	**d18:1/26:1**	N.I.	d18:1/26:1	N.I.	N.I.
			**d20:1/24:1**		
