# Supplementary material for: Ganglioside Profiling of the Human Retina: Comparison with Other Ocular Structures, Brain and Plasma Reveals Tissue Specificities
Source: PLoS One. 2016 Dec 20;11(12):e0168794. doi: 10.1371/journal.pone.0168794 (PMC5173345; doi:10.1371/journal.pone.0168794)
Supplement: S5 Table — We were not able to characterize any molecular species in the minor classes of GQ1b and AcGQ1b. Major molecular species are indicated in bold. N.D.: Non-detected; N.I.: Non-identified. (PDF) [file pone.0168794.s005.pdf]

**S5 Table. Ceramide molecular species of the brain ganglioside classes characterized by HRMS with the LTQ-Orbitrap mass spectrometer.** We were not able to characterize any molecular species in the minor classes of GQ1b and AcGQ1b. Major molecular species are indicated in bold. N.D.: Non-detected. N.I.: Non-identified.

| | GM3 | GM2 | GD3 | AcGD3 | GD2 | GD1a | GD1b | AcGD1b | GT3 | AcGT3 | GT1b | AcGT1b |
|---|---|---|---|---|---|---|---|---|---|---|---|---|
| **32:1** | N.I. | N.I. | N.D. | N.D. | N.D. | N.D. | N.D. | N.D. | N.D. | N.D. | N.D. | N.D. |
| **34:1** | **d18:1/16:0** | d18:1/16:0 | d16:1/18:0<br>**d18:1/16:0** | N.D. | N.I. | N.I. | N.D. | N.D. | N.D. | N.D. | **d18:1/16:0** | N.D. |
| **36:2** | d17:0/19:2<br>**d18:1/18:1** | **d18:1/18:1** | d17:0/19:2<br>d18:2/18:0<br>**d18:1/18:1** | N.D. | **d18:1/18:1** | **d18:1/18:1** | **d18:1/18:1** | **d20:1/16:1** | **d18:1/18:1** | N.D. | N.D. | N.I. |
| **36:1** | d17:0/19:1<br>**d18:1/18:0** | **d18:1/18:0** | **d18:1/18:0** | N.I. | **d18:1/18:0** | **d18:1/18:0** | **d18:1/18:0** | **d20:1/16:0** | **d18:1/18:0** | **d18:1/18:0** | **d18:1/18:0**<br>**d20:1/16:0** | **d20:1/16:0** |
| **38:2** | **d20:1/18:1** | d18:1/20:1<br>**d20:1/18:1** | **d18:1/20:1**<br>**d20:1/18:1** | N.I. | d18:1/20:1<br>**d20:1/18:1** | **d18:1/20:1**<br>**d20:1/18:1** | d18:1/20:1<br>**d20:1/18:1** | N.D. | N.D. | **d18:1/20:1**<br>**d20:1/18:1** | **d20:1/18:1** | N.D. |
| **38:1** | d18:1/20:0<br>**d20:1/18:0** | **d20:1/18:0** | d18:1/20:0<br>**d20:1/18:0** | **d18:1/20:0** | d18:1/20:0<br>**d20:1/18:0** | **d20:1/18:0** | d18:1/20:0<br>**d20:1/18:0** | d18:1/20:0 | **d18:1/20:0** | **d18:1/20:0** | **d20:1/18:0** | N.I. |
| **40:2** | **d18:1/22:1** | N.D. | **d18:1/22:1** | N.I. | N.D. | N.D. | N.D. | N.D. | N.D. | N.D. | N.D. | N.D. |
| **40:1** | **d18:1/22:0** | N.I. | **d18:1/22:0** | **d20:1/20:0** | N.I. | **d20:1/20:0** | **d20:1/20:0** | N.I. | N.D. | N.D. | N.I. | N.I. |
| **41:1** | **d18:1/23:0** | N.D. | N.D. | N.D. | N.D. | N.D. | N.D. | N.D. | N.D. | N.D. | N.D. | N.D. |
| **42:3** | N.D. | N.D. | **d18:1/24:2** | N.D. | N.D. | N.D. | N.D. | N.D. | N.D. | N.D. | N.D. | N.D. |
| **42:2** | **d18:1/24:1** | N.I. | **d18:1/24:1**<br>**d20:1/22:1** | N.D. | N.I. | N.I. | N.I. | N.D. | N.D. | N.D. | N.D. | N.D. |
| **42:1** | **d18:1/24:0** | N.I. | **d18:1/24:0**<br>**d20:1/22:0** | N.D. | N.D. | N.I. | N.I. | N.D. | N.D. | N.D. | N.I. | N.D. |
| **43:2** | N.I. | N.D. | N.D. | N.D. | N.D. | N.D. | N.D. | N.D. | N.D. | N.D. | N.D. | N.D. |
