# Supplementary material for: Ganglioside Profiling of the Human Retina: Comparison with Other Ocular Structures, Brain and Plasma Reveals Tissue Specificities
Source: PLoS One. 2016 Dec 20;11(12):e0168794. doi: 10.1371/journal.pone.0168794 (PMC5173345; doi:10.1371/journal.pone.0168794)
Supplement: S6 Table — Only the main classes of this tissue (GM3, GM2 and GD3) could be characterized. Major molecular species are indicated in bold. N.D.: Non-detected; N.I.: Non-identified. (PDF) [file pone.0168794.s006.pdf]

S6 Table. Ceramide molecular species of the plasma ganglioside classes characterized by HRMS with the LTQ-Orbitrap mass spectrometer. Only the main classes of this tissue (GM3, GM2 and GD3) could be characterized. Major molecular species are indicated in bold. N.D.: Non-detected. N.I.: Non-identified.

	GM3	GM2	GD3
**32:2**	d16:1/16:1	N.D.	
	d17:1/15:1		
	**d18:1/14:1**		**d18:1/14:1**
**32:1**	**d16:1/16:0**	N.D.	**d16:1/16:0**
	**d17:1/15:0**		
	**d18:1/14:0**		**d18:1/14:0**
**34:2**		d16:1/18:1	
	d18:2/16:0		d18:2/16:0
	**d18:1/16:1**	**d18:1/16:1**	**d18:1/16:1**
**34:1**		d16:1/18:1	
	**d18:1/16:0**	**d18:1/16:0**	**d18:1/16:0**
**36:2**	d16:1/20:1		
	d18:2/18:0	d18:2/18:0	d18:2/18:0
	**d18:1/18:1**	**d18:1/18:1**	**d18:1/18:1**
**36:1**	d16:1/20:0		
	**d18:1/18:0**	**d18:1/18:0**	**d18:1/18:0**
**38:2**	d16:1/22:1		
	d18:2/20:0		
	**d18:1/20:1**	**d18:1/20:1**	**d18:1/20:1**
**38:1**	d16:1/22:0		
	**d18:1/20:0**	**d18:1/20:0**	**d18:1/20:0**
**39:1**	d16:1/23:0	N.D.	N.D.
	**d18:1/21:0**		
**40:3**	d16:1/24:2	N.D.	N.D.
	d17:1/23:2		
	d18:2/22:1		
	**d18:1/22:2**		
**40:2**	d16:1/24:1		d16:1/24:1
	d18:2/22:0		d18:2/22:0
	**d18:1/22:1**	**d18:1/22:1**	**d18:1/22:1**
**40:1**	d16:1/24:0	N.I.	d16:1/24:0
	**d18:1/22:0**		**d18:1/22:0**
**41:2**	d18:2/23:0	N.D.	N.D.
	**d18:1/23:1**		
**41:1**	d17:1/24:0	N.D.	d17:1/24:0
	**d18:1/23:0**		**d18:1/23:0**
**42:3**	N.D.	d18:2/24:1	d18:2/24:1
		**d18:1/24:2**	**d18:1/24:2**
**42:2**	d17:0/25:1		
	d18:2/24:0	d18:2/24:0	d18:2/24:0
	**d18:1/24:1**	**d18:1/24:1**	**d18:1/24:1**
**42:1**	**d18:1/24:0**	**d18:1/24:0**	**d18:1/24:0**
**43:2**	d17:0/26:2	N.D.	N.D.
	d18:2/25:0		
	**d18:1/25:1**		
**43:1**	d17:0/26:1	N.D.	N.D.
	**d18:1/25:0**		
